# From Environmental Evidence to Biomarker Selection: A Structured Decision-Support Process for Human Biomonitoring Studies in Contaminated Sites

**DOI:** 10.3390/toxics14070616

**Published:** 2026-07-15

**Authors:** Elisa Bustaffa, Cristina Aprea, Alessandro Barbieri, Alessandro Benassi, Andrea Borghini, Saverio Caini, Piergiuseppe Calà, Filippo Cellai, Amalia Gastaldelli, Francesco Faita, Miriam Levi, Stefano Masi, Simona Mrakic-Sposta, Katia Russo, Anna Solini, Fabrizio Minichilli

**Affiliations:** 1 Institute of Clinical Physiology, National Research Council (IFC-CNR), Via Moruzzi 1, 56124 Pisa, Italy; elisa.bustaffa@cnr.it (E.B.); andrea.borghini@cnr.it (A.B.); amalia.gastaldelli@cnr.it (A.G.); francesco.faita@cnr.it (F.F.); 2 Southeast Wide Area Public Health Laboratory, Occupational and Environmental Toxicology Unit, Operational Headquarter of Siena, Southeast Tuscany Local Health Authority, Strada del Ruffolo 4, 53100 Siena, Italy; cristina.aprea@uslsudest.toscana.it; 3 Hygiene and Public Health Unit, Southern Area (Pisa-Livorno), Department of Prevention, Northwest Tuscany Local Health Authority (ATNO), Via Carlo Forlanini 26, 57025 Piombino, Italy; alessandro.barbieri@uslnordovest.toscana.it; 4 Regional Laboratories, Department of Padua, Regional Environmental Protection Agency Veneto, Via Ospedale Civile 24, 35121 Padua, Italy; alessandro.benassi@arpa.veneto.it; 5 S.S. Molecular and Lifestyle Epidemiology, S.C. Epidemiology of Risk Factors and Lifestyle, Institute for the Study, Prevention, and Oncology Network (ISPRO), Via Cosimo il Vecchio 2, 50139 Florence, Italy; s.caini@ispro.toscana.it; 6 Prevention Department, Azienda USL Toscana Centro, Via S. Salvi 12, 50135 Florence, Italy; piergiuseppe.cala@uslcentro.toscana.it; 7 Clinical Chemical Analysis Laboratory, Department of Diagnostics, Livorno Hospital, USL Toscana Nordovest, Viale Vittorio Alfieri 36, 57124 Livorno, Italy; filippo.cellai@uslnordovest.toscana.it; 8 Epidemiology Unit, Health Management Staff, Azienda USL Toscana Centro, Presidio San Salvi, Edificio 8 (Villa Fabbri), Via di San Salvi 12, 50135 Florence, Italy; miriam.levi@uslcentro.toscana.it; 9 Department of Clinical and Experimental Medicine, Faculty of Medicine, University of Pisa, Via Savi 10, 56126 Pisa, Italy; stefano.masi@unipi.it; 10 Institute of Clinical Physiology of Milan, National Research Council (IFC-CNR), Piazza dell’Ospedale Maggiore 3, 20162 Milan, Italy; simona.mrakicsposta@cnr.it; 11 Experimental Zooprophylactic Institute of Lazio and Tuscany (IZSLT), Via Appia Nuova 1411, 00178 Rome, Italy; katia.russo@izslt.it; 12 Department of Surgical, Medical, Molecular, and Critical Care Pathology, Faculty of Medicine, University of Pisa, Via Roma 67, 56126 Pisa, Italy; anna.solini@unipi.it

**Keywords:** National Priority Contaminated Sites, human biomonitoring, decision-support process, environmental pollution, health effect, biomarker

## Abstract

Human Biomonitoring (HBM) is a key tool for assessing human exposure to environmental contaminants and identifying early biological changes potentially associated with adverse health effects. However, as contaminated sites are characterized by multiple pollutants, exposure pathways and biological targets, a structured decision-support process is required to guide exposure assessment, biomarker selection, and early health-risk evaluation. This manuscript does not report biomonitoring results but describes the methodological process used to design the INSINERGIA_RT HBM study in two contaminated areas of Tuscany, Italy. The study design integrated environmental evidence, toxicological knowledge, epidemiological priorities, biomarker relevance, analytical feasibility, logistical considerations, and public health needs. Instead of proposing a universally applicable framework, the manuscript presents a structured approach that may support the design of HBM studies in similarly complex settings. Biomarkers are organized within a hierarchical model reflecting successive stages of the biological response to environmental exposure. A central feature is a mechanistic, multi-level approach encompassing upstream pathways (oxidative stress and inflammation), early markers of adverse effects and subclinical organ damage, including cardiovascular, renal, and respiratory alterations, and integrative indicators of cumulative biological responses, such as telomere length, epigenetic changes, and metabolomic profiles. Applicability is illustrated through an ongoing HBM study in Livorno and Piombino (Tuscany).

## 1. Introduction

Environmental contamination in both active and decommissioned industrial areas remains a major public health concern worldwide, particularly in regions characterized by complex mixtures of pollutants and long-term, low-dose exposures. Among these, contaminated industrial sites represent a priority in the field of environmental health, as they often result from historical industrial activities and are frequently located near residential urban areas, exposing resident populations to multiple environmental hazards and increasing their vulnerability to adverse health effects [1,2,3,4,5,6,7].

In Italy, National Priority Contaminated Sites (NPCSs) for remediation are defined by the Italian Ministry of Environment and Energy Security as large areas of particular environmental concern, characterized by contamination of multiple environmental matrices and associated with significant health, ecological, and socioeconomic risks. Currently, Italy hosts 42 NPCSs, often characterized by complex mixtures of industrial pollutants affecting air, soil, and water, and involving municipalities hosting major industrial facilities such as petrochemical plants, refineries, port activities and steel production facilities.

In these contexts, traditional environmental monitoring alone often fails to adequately capture the real individual exposure burden, as it does not account for variability in behaviors, exposure pathways, and biological susceptibility. Human Biomonitoring (HBM) has therefore emerged as a key approach to bridge this gap, allowing for the direct assessment of internal exposure through the measurement of chemicals, their metabolites, or biological responses in human matrices. While HBM has historically focused on exposure biomarkers, there is increasing recognition that this approach alone is insufficient to fully understand the health implications of environmental contamination. Exposure biomarkers provide information on the amount of contaminants entering the body but do not capture the biological responses triggered by exposure or the early stages of disease development. For this reason, recent developments in HBM have progressively shifted toward the inclusion of effect biomarkers and, more recently, biomarkers reflecting cumulative and systemic biological responses, such as those related to aging and epigenetic regulation.

Although biomarker selection in HBM studies is generally supported by available scientific evidence, the rationale underlying the integration of different biomarker classes, contaminant prioritization, sampling strategy, and feasibility constraints is not always explicitly documented. This may limit the transparency of the methodological choices underpinning study design and reduce the ability to compare methodological choices across studies conducted in different contaminated-site contexts.

Within this context, the INSINERGIA project (Valutazione dell’esposizione della popolazione a contaminanti persistenti, metalli e PFAS e dei relativi effetti sulla salute, con particolare attenzione alle popolazioni più suscettibili; Assessment of population exposure to persistent contaminants, metals and PFAS and related health effects, with particular attention to the most susceptible populations), funded under the Italian National Recovery and Resilience Plan and its complementary initiatives, aims to develop integrated approaches for evaluating environmental exposures and health effects in populations residing in contaminated NPCSs across Italy. In the Tuscany Region, the INSINERGIA_RT (Regione Toscana, Tuscany Region) study focuses on populations living in the municipalities hosting the NPCSs of Livorno and Piombino, historically characterized by intensive industrial activities such as steel production, refining, port activities and power plants, which have contributed to significant environmental contamination. Epidemiological evidence from the SENTIERI Project (Studio Epidemiologico Nazionale dei Territori e degli Insediamenti Esposti a Rischio di Inquinamento), the Italian national surveillance system for populations residing near contaminated sites, has documented health criticalities in these areas consistent with long-term environmental exposure, reinforcing the need for integrated HBM approaches that combine exposure assessment with early biological effect indicators [8].

The present manuscript addresses this methodological gap by describing and documenting the rationale underlying the design of the INSINERGIA_RT HBM study. Rather than presenting only the final study protocol, it explains how key methodological decisions—including the selection of study sites, target population, biological matrices, contaminants, biomarkers, and analytical strategy—were reached by integrating environmental evidence, toxicological knowledge, epidemiological findings, analytical feasibility, statistical requirements, operational constraints, and public health priorities.

The aim is not to propose a universally applicable decision algorithm, but to provide a transparent, real-world account of the methodological process used to design a population-based HBM study in contaminated sites. Accordingly, the manuscript explicitly reports the rationale underlying each methodological choice, including site selection, contaminant prioritization, biomarker selection, sampling strategy, and population definition, together with the scientific and operational considerations that guided these decisions. The INSINERGIA_RT study conducted in the NPCSs of Livorno and Piombino serves as a real-world case study illustrating this process and provides a practical methodological reference for researchers designing HBM studies in comparable environmental settings.

The novelty of this work does not lie in proposing new biomarkers or in validating a universal HBM model, but in explicitly documenting the methodological reasoning that guided the design of a real-world contaminated-site HBM study. By showing how environmental evidence, toxicological plausibility, epidemiological relevance, analytical feasibility, operational constraints, and statistical requirements were integrated, the manuscript provides a practical reference for researchers facing similar methodological challenges.

In addition, the proposed methodological framework promotes the integration of conventional epidemiological approaches with advanced multi-level analytical strategies to identify composite exposure–response patterns and generate hypotheses for future longitudinal investigations.

## 2. Structured Decision-Support Process for Study Design and Biomarker Selection

The proposed HBM planning process provides a structured, stepwise approach for integrating environmental, toxicological, epidemiological, statistical, and operational considerations regarding study design. The framework follows a flexible sequential process that can be adapted to a wide range of contaminated site settings and supports all major stages of HBM study design, including site characterization, population definition, exposure assessment, sampling strategy, contaminant prioritization, biomarker selection, and the development of epidemiological and statistical analysis plans. Attention is given to the evaluation of temporal variability, biological plausibility, analytical feasibility, and operational constraints. By explicitly documenting the rationale behind each methodological decision, the framework facilitates a comprehensive evaluation of environmental exposures, early biological responses, and potential health effects, while enhancing transparency, consistency, and adaptability across different settings.

### 2.1. Site and Contaminants Selection

Site selection should be guided by both environmental significance and public health relevance. Candidate sites should be evaluated according to documented environmental contamination involving one or multiple pollutant classes, availability of historical environmental monitoring and epidemiological data, evidence or suspicion of chronic population exposure, size and stability of the resident population, feasibility of recruitment and biological sampling, laboratory capacity, available resources, and project timeline. Sites not meeting these criteria may be deprioritized or considered for future dedicated investigations. The final selection should balance scientific priorities, public health needs, analytical feasibility, and operational constraints to identify sites that are environmentally relevant, epidemiologically informative, and suitable for population-based HBM investigations.

Contaminant selection and prioritization should be based on the integration of environmental monitoring data, toxicological knowledge, epidemiological evidence, and analytical feasibility. Priority may be assigned to contaminants characterized by environmental persistence, widespread occurrence, bioaccumulation potential, biological activity, and documented or suspected associations with adverse health outcomes. Particular attention should be given to contaminants capable of inducing common biological pathways, such as oxidative stress, inflammation, endocrine disruption, metabolic alterations, or other mechanisms relevant to the study objectives. The prioritization process should also consider the availability of validated analytical methods, expected exposure levels, detection frequencies, resource requirements, and the relevance of contaminants to local environmental conditions. Contaminants may be excluded when environmental evidence is insufficient, expected exposure levels are negligible, toxicological information is limited, or analytical feasibility is incompatible with study objectives and available resources.

This process aims to identify a contaminant panel that supports an integrated assessment of environmental exposures and their potential biological and health consequences.

### 2.2. Communication Strategy and Community Engagement

Effective communication and community engagement are essential components of HBM studies, particularly when conducted in contaminated sites where environmental concerns and risk perception may influence participation and trust. Communication strategies should promote transparency, support informed participation, and foster dialogue among researchers, public health authorities, stakeholders, and local communities.

Communication activities may include the dissemination of study information through institutional channels, public meetings, community organizations, healthcare professionals, traditional media, and digital platforms. Importance should be given to the use of clear, accessible, and scientifically accurate language adapted to different audiences.

Community engagement should be considered throughout the study process, from study planning and recruitment to the communication of results. The involvement of local stakeholders, healthcare providers, and community representatives may contribute to improving participation rates, addressing concerns related to environmental exposures, and enhancing the social relevance and acceptability of the study.

A structured communication strategy can support recruitment, strengthen public trust, improve understanding of study objectives and limitations, and facilitate the translation of scientific findings into public health actions.

### 2.3. Epidemiological Design and Analytical Strategy

Selecting an appropriate epidemiological design and analytical strategy is a critical step in HBM study planning and should be guided by the study objectives, characteristics of the exposure scenario, expected biological responses, and available resources. Different epidemiological designs may be considered, including cross-sectional, longitudinal, panel, cohort, or repeated-measure approaches. The selected design should reflect the temporal pattern of environmental exposures, the expected timing of biological responses, and the feasibility of participant follow-up.

Similarly, statistical models should be selected according to the nature of the exposure indicators, biomarker distributions, and study outcomes. Continuous biomarkers may be evaluated using linear regression models or mixed-effects models when repeated measurements are available, whereas categorical outcomes may be analyzed using logistic regression models. Multivariable approaches should account for relevant confounding factors, including demographic, socioeconomic, occupational, behavioral, and clinical characteristics. For studies involving multiple biomarkers, high-dimensional datasets, or omics technologies, advanced analytical approaches such as dimension-reduction techniques, machine-learning methods, network analyses, or exposome-oriented models may be considered. The analytical strategy should be defined a priori and aligned with the hierarchical structure of the biomarker framework, supporting the integrated evaluation of environmental exposures, biological responses, and early indicators of health effects.

#### 2.3.1. Definition and Prioritization of Study Endpoints

The definition of study endpoints represents a fundamental step in the design of HBM studies and should be guided by the study objectives, the characteristics of the environmental exposures under investigation, biological plausibility, and available scientific evidence.

Endpoints may be organized according to different levels of the exposure–response continuum. Primary endpoints may include biomarkers of internal exposure and early biological responses, directly linked to the contaminants of concern. Secondary endpoints may include biomarkers reflecting subclinical alterations in target organs or physiological systems, such as cardiovascular, renal, respiratory, metabolic, neurological, or endocrine functions. Exploratory endpoints may include integrative biomarkers and omics-based approaches capable of identifying novel biological pathways, cumulative biological responses, and previously unrecognized exposure-health relationships.

The prioritization of endpoints should consider scientific relevance, expected sensitivity to environmental exposures, analytical robustness, temporal stability, and feasibility within the study setting. Whenever possible, endpoints should be selected according to a hierarchical framework that facilitates the interpretation of relationships between environmental exposures, biological responses, and early indicators of adverse health effects.

This approach supports the development of coherent analytical strategies and promotes an integrated assessment of environmental exposures and their potential impacts on human health.

#### 2.3.2. Biomarker Selection: Mechanistic Multi-Level Framework

Biomarker selection should follow a mechanistic multi-level framework that captures successive stages of the biological continuum connecting environmental exposure to early health outcomes. Biomarkers should be selected according to criteria including biological plausibility, sensitivity to environmental exposures, clinical relevance, invasiveness level of sample collection, analytical robustness, temporal stability, and feasibility in population-based studies. The biomarker panel may be organized into hierarchical levels representing different stages of the biological response. The first level includes biomarkers of upstream biological processes, such as oxidative stress, inflammation, endocrine disruption, or other pathways potentially activated by environmental contaminants. The second level includes biomarkers reflecting early or subclinical alterations in specific target organs or physiological systems, such as cardiovascular, renal, respiratory, metabolic, or neurological functions. The third level comprises integrative biomarkers reflecting cumulative biological responses and long-term adaptation processes, including telomere length, epigenetic modifications, metabolomic profiles, and other omics-based indicators.

Rather than presenting separate study objectives, these biomarkers should be regarded as complementary components of an integrated biological framework. Their hierarchical organization supports the interpretation of exposure–response relationships and facilitates a comprehensive assessment of the potential early biological and health effects associated with environmental exposures.

The final biomarker panel should be adapted to the specific environmental context, contaminants of concern, study objectives, available resources, and analytical capacities.

#### 2.3.3. Study Population and Sampling Strategy

The study population and sampling strategy should be defined to balance representativeness, comparability, statistical power, and operational feasibility. Population eligibility criteria should be established according to the study objectives, the characteristics of the environmental exposure scenario, and the need to minimize major sources of confounding.

Different sampling approaches may be considered, including cross-sectional, longitudinal, panel, or repeated-measure designs. The choice should be guided by scientific objectives, available resources, expected participation rates, logistical constraints, and the temporal characteristics of the exposure and health outcomes under investigation.

Sampling strategies should ensure adequate representation of relevant population subgroups, such as age classes, sex, socioeconomic categories, or exposure strata, while maintaining sufficient statistical power for planned analyses. Particular attention should be given to potential sources of selection bias and to the representativeness of the target population.

Exposure characterization should integrate, whenever possible, environmental monitoring data, emission inventories, atmospheric dispersion patterns, prevailing wind directions, land-use information, and other site-specific determinants. In settings where detailed environmental information is limited, indirect exposure indicators, including residential distance from contaminated sites or potential emission sources, may be considered as proxy measures of environmental exposure.

The final sampling design should provide a balance between methodological rigor, analytical feasibility, and the ability to evaluate relationships between environmental exposures, biological responses, and early indicators of health effects.

#### 2.3.4. Definition of Inclusion and Exclusion Criteria

Inclusion and exclusion criteria should be defined according to the study objectives, the characteristics of the exposure scenario, the target population, and the biological endpoints under investigation. Inclusion criteria should ensure that participants are representative of the population of interest and have a sufficient probability of experiencing the environmental exposures being assessed.

Attention should be given to factors influencing exposure duration, exposure intensity, and the biological relevance of the selected biomarkers. Depending on the study objectives, eligibility criteria may consider age range, residential history, occupational status, duration of residence, and other factors relevant to exposure assessment.

Exclusion criteria should aim to minimize potential sources of bias and reduce the influence of conditions that could substantially alter biomarker levels independently of environmental exposures. These may include severe acute illnesses, conditions affecting biological sample collection, inability to provide informed consent, or other factors that could compromise data quality or participant safety.

The final definition of inclusion and exclusion criteria should balance scientific validity, representativeness of the target population, ethical considerations, and operational feasibility.

#### 2.3.5. Recruitment and Enrolment Strategy

The recruitment and enrolment process represents a critical component of HBM studies, as it directly influences participation rates, representativeness, and the overall validity of study findings. Recruitment strategies should be selected according to the characteristics of the target population, available resources, local context, and study objectives.

Different recruitment approaches may be adopted, including population registries, healthcare databases, community-based recruitment, institutional networks, or mixed strategies. The selected approach should aim to maximize participation while minimizing selection bias and ensuring equitable access to study participation.

The enrolment process should include procedures for eligibility verification, informed consent collection, biological sample collection, administration of questionnaires, and the acquisition of clinical or instrumental measurements when required. Standardized operating procedures should be adopted to ensure consistency across study sites and reduce potential sources of measurement error.

Attention should be given to participant burden, accessibility of study procedures, protection of personal data, and ethical considerations. Strategies to improve participation, such as clear communication, flexible scheduling, involvement of healthcare professionals, and community engagement activities, should be considered whenever appropriate.

A well-designed recruitment and enrolment strategy contributes to improving data quality, representativeness of the study population, and the reliability of the assessment of environmental exposures and their potential biological and health effects.

#### 2.3.6. Case Report Forms, Data Management, and Privacy Protection

The collection and management of individual-level information should follow standardized procedures to ensure data quality, consistency, traceability, and compliance with ethical and regulatory requirements. Case Report Forms (CRFs) and questionnaires should be developed to collect information relevant to exposure assessment, health status, lifestyle factors, occupational history, residential history, and other variables required for epidemiological analyses and interpretation of biomonitoring results. Whenever possible, validated questionnaires and standardized coding systems should be adopted to improve data comparability and facilitate integration across studies. Data collection procedures should include quality assurance measures such as staff training, standardized operating procedures, automated consistency checks, and periodic data verification.

A comprehensive data management plan should be established prior to study implementation. This plan should define procedures for data collection, storage, access control, validation, cleaning, documentation, and long-term archiving. Particular attention should be given to the integration of questionnaire data, environmental measurements, biomonitoring results, and clinical information within a secure and traceable data infrastructure.

The protection of participants’ privacy and personal data should be ensured through pseudonymization or anonymization procedures, restricted access to identifiable information, secure data storage systems, and compliance with applicable ethical and legal requirements, including data protection regulations. Data governance procedures should clearly define responsibilities for data access, sharing, retention, and secondary use.

The adoption of standardized CRFs, robust data management procedures, and appropriate privacy safeguards supports the generation of reliable, reproducible, and ethically sound evidence for environmental health research and public health decision-making.

#### 2.3.7. Definition of Questionnaires

The collection of individual-level information represents a fundamental component of HBM studies and should be planned according to the study objectives, contaminants of interest, and anticipated biological outcomes. Standardized questionnaires should be used to collect information necessary for exposure assessment, identification of potential confounding factors, and interpretation of biomarker measurements.

The selection of questionnaire domains should be guided by biological plausibility and known determinants of exposure and health outcomes. Questionnaire sections may include sociodemographic characteristics, residential history, occupational exposures, lifestyle factors, dietary habits, smoking and alcohol consumption, physical activity, medical history, medication use, and other factors relevant to the contaminants and biomarkers under investigation.

Whenever possible, validated questionnaires should be preferred to improve data quality, comparability, and reproducibility. The level of detail collected should balance scientific objectives, participant burden, data quality, and operational feasibility.

The integration of questionnaire data with environmental measurements, biomonitoring results, and health-related biomarkers supports a more comprehensive assessment of environmental exposures and their potential early biological and health effects.

#### 2.3.8. Statistical Analysis Framework

The analytical strategy should be structured according to the following five main objectives:

- characterization of internal exposure levels to priority contaminants;
- evaluation of the associations between exposure biomarkers and early biological response markers;
- assessment of the relationship between exposure indicators and biomarkers of subclinical effects;
- exploration of integrative biomarkers of cumulative biological stress, including telomere length, DNA methylation, and metabolomic profiles;
- identification and characterization of composite multi-level exposure–response patterns linking environmental contaminants, biomarkers of exposure, intermediate biological responses, molecular markers of susceptibility, and early health effects.

The statistical analysis framework should be tailored to fit the study objectives, endpoint definition, biomarker characteristics, and complexity of the exposure scenario. Analyses should enable an integrated evaluation of environmental exposures, biomarkers of internal dose, early biological responses, and indicators of subclinical health effects.

The analytical strategy should include descriptive analyses to characterize the study population and biomarker distributions, followed by multivariable models to investigate exposure–response relationships. Statistical methods should be selected according to the type and distribution of outcome variables and may include linear, logistic, generalized linear, or mixed-effects models when appropriate.

Potential confounding factors should be identified *a priori* based on biological plausibility and existing evidence and may include demographic, socioeconomic, occupational, behavioral, dietary, and clinical variables.

Procedures for handling missing data, values below the limits of detection or quantification, outliers, and multiple testing should be specified a priori. For studies involving multiple contaminants, complex exposure mixtures, or high-dimensional biomarker datasets, advanced analytical approaches such as dimension-reduction techniques, mixture models, machine-learning methods, network analyses, or exposome-oriented approaches may be considered.

The statistical analysis plan should be defined before data analysis and documented transparently to ensure consistency between study objectives, endpoint definitions, and analytical methods.

#### 2.3.9. Ethics and Dissemination

Ethical considerations are integral to all phases of HBM studies, from study design and implementation to data analysis and dissemination. All study procedures should be conducted in accordance with applicable ethical principles, national regulations, and international guidelines governing research involving human participants.

Participants should receive clear and comprehensive information regarding study objectives, procedures, potential risks and benefits, biological sample collection, data management practices, and the intended use of study findings. Participation should be voluntary and based on documented informed consent.

Particular attention should be given to the communication of individual and aggregate results. Dissemination strategies should aim to provide accurate, understandable, and scientifically balanced information while avoiding unnecessary alarm or misinterpretation of findings. When appropriate, participants may be offered access to individual biomonitoring results accompanied by adequate contextual information to support their interpretation.

Study findings should be disseminated through scientific publications, technical reports, institutional communication channels, stakeholder engagement activities, and community outreach initiatives. The dissemination process should promote transparency, support public health decision-making, and facilitate the translation of scientific evidence into prevention and risk-management actions.

Whenever possible, data sharing and dissemination activities should follow principles of scientific integrity, transparency, reproducibility, and responsible research practices while ensuring the protection of participant confidentiality and personal data.

## 3. Application of the Structured Decision-Support Process

### 3.1. Case Studies of Livorno and Piombino National Priority Contaminated Sites

The applicability of the proposed framework is illustrated through its implementation in two NPCSs located in Tuscany, Italy: Livorno and Piombino. Table 1 summarizes the decision-making process and the rationale underlying the methodological choices adopted for the two Tuscan NPCSs. Both sites are characterized by a long history of industrial activities, including refining, steel production, energy generation, port operations, and waste management, resulting in complex environmental contamination involving multiple pollutant classes and exposure pathways.

**Table 1:** Structured decision-support process adopted for the INSINERGIA_RT Human BioMonitoring Study.

Alternative Options	Decision-Making Considerations	Final Decision
1. Site and contaminant selection		
National Priority Contaminated Sites (NPCSs)	Environmental contamination profile; epidemiological evidence (see Supplementary Material); public health relevance; resident population size; recruitment feasibility; available resources; project timeline	NPCSs of Livorno and Piombino
All contaminants detected in the study areas (see Supplementary Material)	Environmental monitoring data; persistence; toxicological relevance; expected human exposure; public health concern	PFAS, heavy metals, PCBs, PAHs, VOCs, DL compounds
2. Communication strategy and communication engagement		
Passive information; institutional communication; active community engagement	Transparency; participant trust; informed participation; recruitment effectiveness	General practitioners, institutional communication, press releases, posters, public meetings
3. Epidemiological Design and analytical strategy 3.1. Study design and Prioritization of endpoints		
Cross-sectional; longitudinal cohort; repeated-measures; panel study	Scientific objectives; statistical power; available resources; feasibility; project duration	Cross-sectional HBM study
Exposure biomarkers; early-effect biomarkers; clinical outcomes; clinical integrated biological approach	Exposure–response continuum; biological plausibility; early detection; study objectives	Primary (exposure), Secondary (effect biomarkers), Exploratory (omics) endpoints
3.2. Biomarker selection and treatment and analyses of biological samples		
Wide panel of candidate biomarkers identified from literature	Toxicological and epidemiological evidence (SENTIERI; see Supplementary Material and the text); analytical robustness; invasiveness level; laboratory availability; temporal stability; sample volume; costs; participant burden	Final panel of exposure, cardiovascular, renal, respiratory, inflammatory, oxidative stress, aging and metabolomic biomarkers
Blood; urine; hair; nails; saliva	Suitability for prioritized biomarkers; analytical validation; contaminant characteristics; feasibility	Blood, urine, and saliva
3.3. Study populations and sampling strategy		
Children; adults; elderly	Scientific objectives; residential stability; biological variability; ethical aspects; operational feasibility	Adults aged 20–64 years
Alternative sample sizes	Statistical power; available budget; human resources; project timeline	1000 participants (700 Livorno; 300 Piombino)
For exposure assessment: dispersion modeling; personal monitoring; GIS models; distance-based approach	Availability of environmental data; feasibility; comparability	Four distance-based exposure classes
For sampling strategy: simple random sampling; convenience sampling; stratified sampling	Population representativeness; comparability; statistical robustness	Stratified sampling by sex, age and exposure class
3.4. Definition of inclusion and exclusion criteria		
Inclusion: different residence periods; unrestricted age	Chronic exposure assessment; bioaccumulation; exposure misclassification; scientific and ethical considerations	Residence ≥ 5 years; age 20–64 years
Exclusion: No exclusions; disease-specific exclusions	Biomarker interpretation; participant safety; validity of measurements	Pregnancy; inability to consent; conditions preventing sampling
3.5. Recruiting and enrolment		
Mail invitation; public campaign; GP invitation; telephone contact	Recruitment efficiency; available time; project timeline	Telephone invitation
Alternative visit sequences	Duration of procedures; participant burden; sample integrity; operational efficiency	Visit structure reported in Figure 1
3.6. REDCap eCase Report Form		
Excel; Access; REDCap	GDPR compliance; audit trail; quality control; multicentre management	REDCap electronic CRF
3.7. Questionnaire		
Newly developed questionnaire; validated questionnaires; adapted questionnaire	Comparability with previous studies; completeness of information; feasibility	Structured questionnaire (~80 items) adapted from previous epidemiological studies
3.8. Epidemiological and statistical analysis		
Descriptive analyses only; standard regression; multilevel models; omics integration	Study objectives; outcome type; confounding control; exposure–response evaluation; interpretability	Multivariable regression models; exposure–response analyses; stratified and sensitivity analyses; integration with metabolomic and omics data where appropriate
4. Ethics and dissemination		
Standard informed consent only; active dissemination strategy	Transparency; participant engagement; public health relevance; regulatory compliance	Ethics approval, informed consent, dissemination to participants, institutions and stakeholders

Legend—PFAS: Per- and PolyFluoroalkyl Substances; PCBs: PolyChlorinated Biphenyls; PAHs: Polycyclic Aromatic Hydrocarbons; VOCs: Volatile Organic Compounds; DL: Dioxin-Like; HBM: Human BioMonitoring.

**Figure 1:**
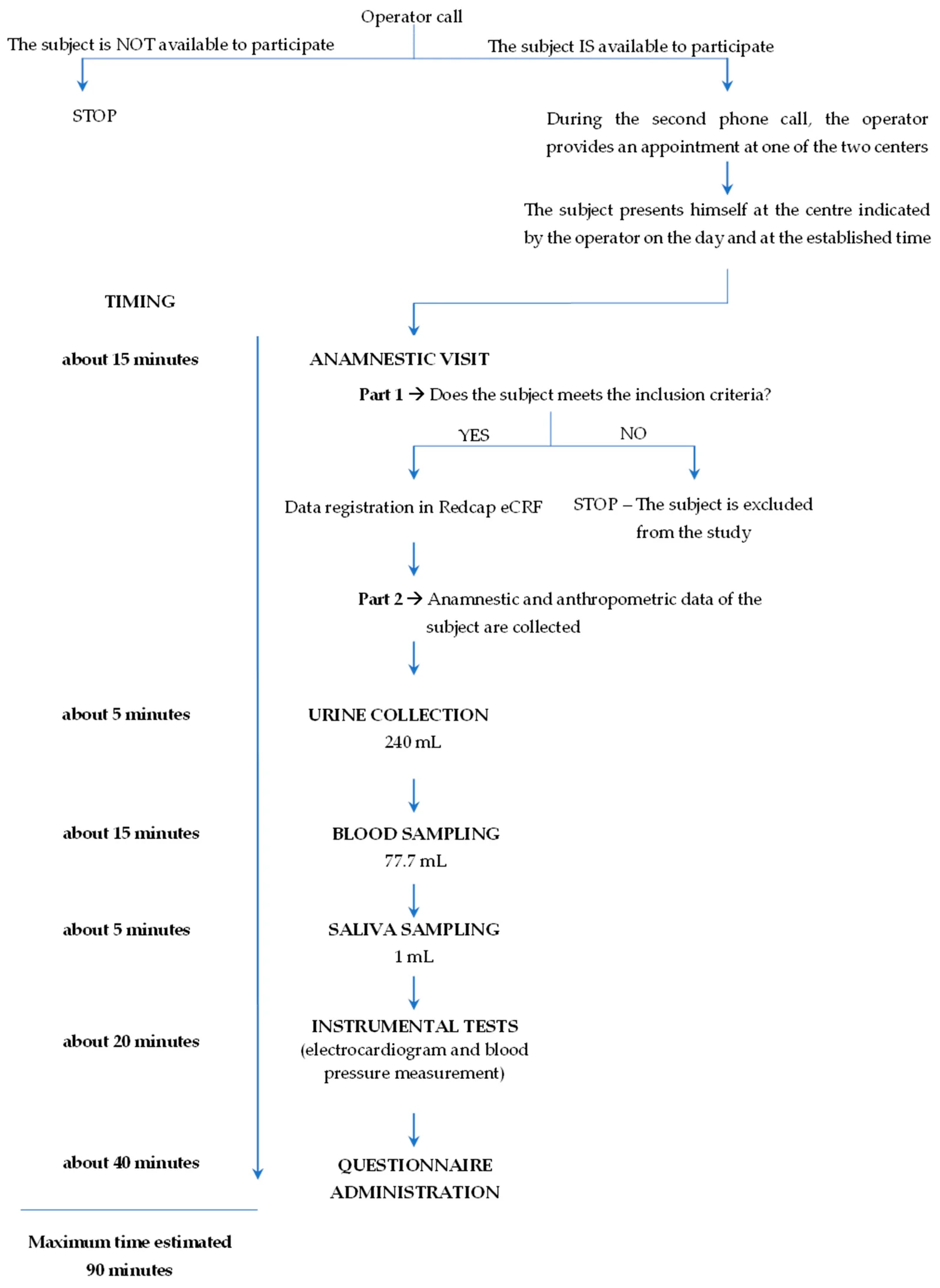
Diagram showing the various phases of recruitment and enrolment, the interventions planned for the enrolled subjects and the related timeframes.

Environmental investigations conducted within the two NPCSs have documented contamination of soil, groundwater, and other environmental matrices by metals, hydrocarbons, polycyclic aromatic hydrocarbons (PAHs), chlorinated solvents, and other industrial pollutants. Epidemiological evidence from the SENTIERI surveillance system has reported excesses of selected adverse health outcomes, including cancer, cardiovascular diseases, digestive diseases, and congenital anomalies, supporting the relevance of these areas for human biomonitoring studies.

The two NPCSs were selected because they provide a well-characterized setting with multiple exposure sources, extensive environmental and epidemiological data, and resident populations suitable for population-based HBM research.

Detailed descriptions of site characteristics, contamination profiles, industrial history, and available epidemiological evidence are provided in Supplementary Material.

Alternative contaminated sites within Tuscany were considered during the planning phase. Livorno and Piombino were ultimately prioritized because they offered the most appropriate balance between environmental complexity, availability of environmental and epidemiological evidence, public health relevance, resident population size, feasibility of recruitment, and compatibility with the INSINERGIA project timeline and resources. Other sites were not excluded because of lower scientific relevance, but because they did not offer the same combined feasibility for implementing a population-based HBM study within the available operational constraints.

### 3.2. Application of Communication Strategy and Community Engagement

To promote informed participation and ensure transparency, a structured communication strategy will be implemented among residents of the study areas. Information will be disseminated through institutional channels, press releases, social media, and posters displayed in healthcare facilities and municipal information points. Public events and local meetings will also be organized to illustrate the study objectives, procedures, and expected benefits, with particular attention to using clear and accessible language. A key component of the strategy is the active involvement of general practitioners, who were informed in advance about the project so they can address citizens’ questions and facilitate their participation. The overall goal is to enhance public trust, improve environmental health awareness, and support participant recruitment (Table 1).

### 3.3. Application of the Epidemiological Design and Analytical Strategy

The INSINERGIA_RT study is a non-profit, cross-sectional HBM study with minimal intervention, involving biological sample collection, questionnaire administration, and clinical measurements. Data collection will be performed within 16 months (May 2025–December 2026), including protocol development, submission and approval to the Ethics Committee, and active project management (Table 1).

Alternative designs, including longitudinal cohorts, panel studies, and repeated-measure approaches, were considered during protocol development. However, given the available funding, project duration, expected recruitment capacity, laboratory workload, and the objective of obtaining a broad characterization of environmental exposure in the target population, a cross-sectional design provided the best balance between scientific validity, statistical power, participant burden, and operational feasibility (Table 1).

Given its cross-sectional design, causal associations between outcomes and exposure markers cannot be established in this study. The analyses will therefore focus on identifying associations between exposure indicators, biomarkers of internal dose, and early biological effect markers. Any interpretation of exposure–response patterns will be made cautiously and considering the limitations inherent to cross-sectional studies (Table 1).

#### 3.3.1. Endpoints

To improve the transparency and coherence of the analytical framework, study endpoints were hierarchically organized along the exposure–response continuum (Table 1). As shown in Table 2, endpoints were classified into primary, secondary, and exploratory categories, reflecting their role in characterizing environmental exposures, identifying early biological responses and subclinical health effects, and exploring integrative biological mechanisms potentially involved in exposure-related health outcomes (Table 2).

#### 3.3.2. Rationale for Biomarker Selection, Treatment and Analyses of Biological Samples

The biomarker panel was selected using three main criteria:

1. environmental contamination patterns documented in the Livorno and Piombino NPCSs, where Per- and Polyfluoroalkyl Substances (PFAS), heavy metals, PolyChlorinated Biphenils (PCBs), PAHs, Volatile Organic Compounds (VOCs) and dioxin-like compounds have been repeatedly detected in soil, water and air;
2. toxicological evidence indicating their bioaccumulation, metabolic activation and capacity to induce systemic biological damage, including endocrine disruption, oxidative stress, inflammation, metabolic impairment, cardiovascular and renal dysfunction and accelerated biological aging;
3. epidemiological literature and the HBM4EU project recommendations (https://www.hbm4eu.eu/hbm4eu-substances/ (accessed on 15 May 2026), which identify these substances as priority contaminants due to their prevalence in industrial regions and their established or suspected health risks.

Additional candidate biomarkers were evaluated during protocol development. The final biomarker panel was selected by integrating methodological, toxicological, and epidemiological considerations, balancing analytical robustness, biological plausibility, temporal stability, laboratory availability, sample volume requirements, participant burden, available resources, and feasibility within a large population-based study (Table 1). The final selection was further tailored to the environmental and epidemiological profile of the investigated NPCSs. Previous surveillance activities and findings from the SENTIERI Project consistently reported excess risks for cardiovascular and respiratory diseases, supporting the prioritization of biomarkers related to cardiovascular and respiratory pathways. Renal biomarkers were additionally included because of the well-established nephrotoxic potential of several contaminants of concern, including metals and PFAS (Table 1). This strategy ensured consistency between site-specific environmental exposures, epidemiological evidence, and the biological mechanisms targeted by the study. Although biomarkers of neurotoxicity were not included in the core panel, the establishment of the INSINERGIA_RT Biobank will enable future investigations of neuronal biomarkers, including acetylcholinesterase activity, as new research questions may arise (Table 1). The resulting biomarker panel enables an integrated assessment of internal exposure, early biological effects, and potential health effects.

The selected biomarkers (Table 3) include exposure biomarkers, cardiovascular and renal biomarkers, respiratory disease biomarkers, and biomarkers associated with disease risk.

In addition to contaminant-specific biomarkers, the study will analyze a set of routine clinical biochemical parameters (e.g., glucose, lipid profile, liver and kidney function markers) to support the interpretation of biological responses and strengthen the control of potential confounders. The analytical panel also includes biomarkers of biological aging, such as indices of oxidative stress, inflammation, and cellular senescence, which are increasingly recognized as sensitive indicators of cumulative environmental stress. Furthermore, targeted and untargeted metabolomic profiling will be performed on selected biological samples. This approach will enable the detection of global metabolic perturbations linked to chronic exposures typical of contaminated industrial sites and supports the discovery of novel biomarkers of exposure and effect.

To complement the biochemical and molecular analyses, the study incorporates instrumental assessments aimed at evaluating early functional changes in organ systems potentially affected by environmental contaminants. Specifically, each participant will undergo a standardized blood pressure measurement (following international guidelines) and a resting ElectroCardioGram (ECG). These measurements contribute to the identification of subclinical cardiovascular alterations, which are particularly relevant in populations exposed to pollutants known to affect cardiac rhythm, vascular function, and autonomic regulation.

The integration of exposure biomarkers, early-effect biomarkers, clinical parameters, biological aging indicators, metabolomic data, and instrumental measurements will provide a multidimensional assessment of the health impact of living in contaminated industrial areas.

All sample processing procedures, storage, and analysis methodologies are detailed in Supplementary Material (Tables S1–S8). Samples will be transported in secondary and third-party boxes and equipped with a GPS data logger to ensure temperature control and traceability.

Blood and urine were selected as the primary biological matrices because together they allow the assessment of both persistent bioaccumulative contaminants and non-persistent compounds, while also supporting the measurement of exposure biomarkers, early-effect biomarkers, and routine clinical parameters (Table 1). Alternative matrices, including hair, nails, saliva, and other non-invasive specimens, were considered but not selected because they provided more limited information for the target contaminants, lower analytical standardization within the participating laboratories, or weaker comparability with existing HBM evidence (Table 1). The term “blood” refers to different biological matrices depending on the analytical application. Whole blood, serum, or plasma were selected according to the analytical requirements of each biomarker, established laboratory protocols, and assay validation procedures (Tables S1–S8). Detailed information on sample collection tubes, processing procedures, aliquoting and storage conditions is reported in the Supplementary Material.

Aliquots not used for immediate analysis will be stored in the study’s dedicated Biobank.

Repeated biological sampling was considered for biomarkers characterized by substantial temporal variability. However, because of the large planned sample size, participant burden, project timeline, laboratory capacity, and available resources, a single biological sampling session was adopted. To minimize and account for short-term variability, standardized procedures for sample collection, processing, transport, and storage were implemented, and information on recent exposures, diet, smoking, medication use, and other lifestyle factors will be collected through questionnaires and considered in the statistical analyses.

Table 3 reports the application of the Structured Decision-Support Process to biomarker selection.

The following sections describe in detail the rationale for biomarker selection, the procedures for processing and the analysis methodology for the biomarkers and indicators selected.

##### Biomarker of Exposure

To identify exposure biomarkers, as specified at the beginning of Section 3.3.2, the environmental contamination patterns documented in the Livorno and Piombino NPCSs where PFAS, heavy metals, PCBs, PAHs, VOCs and dioxin-like compounds have been repeatedly detected in soil, water and air, were considered (Table 1). These classes of compounds were also chosen based on their well-documented effects on human health, as reported in the Supplementary Material [9,10,11,12,13,14,15,16,17,18,19,20,21,22,23,24,25,26,27,28,29,30,31,32,33,34,35,36,37,38,39,40,41,42,43,44,45,46,47,48,49,50,51,52,53,54,55,56,57,58,59,60,61,62,63,64,65,66,67,68,69,70,71,72,73,74,75,76,77,78,79,80,81,82,83,84,85,86,87,88,89,90,91,92,93,94,95]. In particular, Dioxin-Like Polychlorobiphenyls (number of substances that will be analyzed n = 12), Non-Dioxin-Like Polychlorobiphenyls (n = 6), OrganoChlorine Pesticides (n = 21), OrganoPhosphorus Pesticides (n = 53), PolyChlorinated Dibenzo-p-Dioxins and DibenzoFurans (n = 17), PFASs (n = 11) and copper will be measured in serum, and chromium and lead will be measured in whole blood (Table 3; Table S1 lists the specific substances that will be measured in serum) (total n = ~120) [96,97]. All samples will be treated according to the methods described in Table S1 until they are transferred to the specific analysis laboratories where they will be analyzed according to the methods reported in Table 3 and described in detail in Table S1.

The exposure biomarkers identified as characteristic of the study areas and which will be measured in urine (n = 35) are VOCs, PAHs, arsenic, mercury and some heavy metals (Table 3). Specifically, for PAHs, the concentration of 1-Hydroxypyrene, considered the gold-standard biomarker of PAHs exposure, will be measured in urine [98,99,100,101]. All samples will be treated according to the methods described in Table S2 until they are transferred to the specific analysis laboratory where they will be analyzed according to the methods reported in Table 3 and described in detail in Table S2.

##### Biochemical Analytes

The most relevant analytes (n = 62) were selected in association with the expected exposure and their reliability in providing accurate information about the subjects’ health risk profile (Table S3) given their ability to characterize metabolic, hepatic, renal and cardiovascular function, support the interpretation of exposure and effect biomarkers and identify confounders and comorbidities in exposed populations (Table 1).

Several laboratory parameters were included because of their established clinical relevance to assess the subjects’ health risk profile, measuring some specific markers that will also be performed in this study. Some of these markers are:

- cardiovascular markers: lipid profile, which plays an important role in cardiovascular disease prevention [102,103] (total cholesterol, triglycerides and high-density lipoprotein), complete blood count which identifies potential issues like anemia which impacts oxygen supply to the heart and, homocysteine, an amino acid that, when high, acts as a marker of coronary and peripheral vascular disease [104];
- renal markers: serum creatinine, a waste product from muscle metabolism filtered by the kidneys. (high levels often indicate kidney damage), urea and uric acid (elevated levels may indicate kidney stones or gout) and electrolytes such as sodium and potassium and chloride (damaged kidneys struggle to balance these electrolytes) [105];
- metabolic markers: indicate how efficiently the body converts food into energy, focusing on sugar regulation, fat processing, and inflammation. Key markers include fasting plasma glucose (measures current blood sugar), glycated hemoglobin (provides a 3-month average, indicating long-term sugar management and risk of diabetes), and insulin (measures how much insulin is required to manage blood sugar) [104];
- liver markers: elevated Liver enzymes (like alanine aminotransferase and aspartate aminotransferase) can indicate non-alcoholic fatty liver disease, a condition strongly linked to insulin resistance; other markers such as conjugated (direct) bilirubin, lactate dehydrogenase, total protein, globulins, and albumin can help determine the area of hepatic injury [106].

Furthermore, these analytes will also be essential for defining the biomarkers of biological effect described in the following paragraphs.

All samples will be treated according to the methods described in Table S3 until they are transferred to the specific analysis laboratory where they are analyzed according to the methods reported in Table 3 and described in detail in Table S3.

##### Biomarkers of Cardiovascular and Renal Risk

The selection of high-sensitivity C-reactive protein, interleukin 6, 1β, 18, tumor necrosis factor, and monocyte chemoattractant protein-1 is grounded in their central role as key mediators and integrators of systemic inflammatory responses. Pro-inflammatory cytokines such as interleukin-1β and tumor necrosis factor act as upstream regulators that trigger cytokine cascades and endothelial activation, while interleukin 6 drives hepatic acute-phase protein synthesis (including C-reactive protein), making high-sensitivity C-reactive protein a robust downstream, stable marker of low-grade chronic inflammation. Monocyte chemoattractant protein-1 reflects monocyte recruitment and vascular inflammatory activation, and interleukin 18 is implicated in inflammasome signaling and chronic immune activation. Collectively, these biomarkers provide a comprehensive assessment of innate immune activation, cytokine signaling, and chronic systemic inflammation, all of which are known to be influenced by environmental and occupational exposures (Table 1).

The inclusion of F2-isoprostanes, 3-nitrotyrosine, thiobarbituric acid reactive substances, and 4-hydroxynonenal is justified by their ability to reflect oxidative damage to lipids and proteins, a key mechanistic pathway linking environmental contaminants to disease. F2-isoprostanes, derived from arachidonic acid peroxidation, are considered among the most reliable in vivo markers of lipid peroxidation due to their chemical stability and specificity. Thiobarbituric acid reactive substances (largely reflecting malondialdehyde), 4-hydroxynonenal, and related aldehydes are secondary products of lipid peroxidation that form adducts with biomolecules, while 3-nitrotyrosine indicates nitrosative stress and protein modification. These biomarkers are widely used in epidemiological studies because they provide integrated measures of oxidative injury downstream of reactive oxygen and nitrogen species generated by pollutant exposure (Table 1).

Combined assessment of inflammatory and oxidative stress biomarkers allows evaluation of the “oxidative stress–inflammation axis”, a key mechanism linking environmental pollution to cardiovascular and renal disease [107,108,109].

Cardiovascular health assessment will also be based on analysis of inflammatory status and oxidative stress performed on peripheral blood (Table 1). Furthermore, markers of incipient renal damage such as cystatin C, beta-2 microglobulin (blood) and albuminuria, creatininuria and their ratio, kidney injury molecule 1, neutrophil gelatinase-associated lipocalin, beta-2 microglobulin (urine) will be measured (Table S4).

Blood and urine samples—Samples will be treated according to the methods described in Table S4 until they are transferred to the specific analysis laboratories where they will be analyzed according to the methods reported in Table 3 and described in detail in Table S4.

Non-Invasive Biomedical Devices—A 12-lead ECG will be recorded as an indicator of subclinical cardiac damage resulting from exposure to cardiovascular risk factors (Table 1). Artificial intelligence analyses can be performed on the electrocardiographic tracings acquired using software internally validated at the Mayo Clinic to estimate actual cardiovascular age. The gap with the participant’s chronological age could provide information on premature aging of the cardiovascular system resulting from environmental exposure to contaminants and also allows for the definition of the potential patient prognosis. In the two study centers, two workstations (one for ECG signal acquisition and the other for central and branchial blood pressure signal acquisition) will be set up. All cardiovascular readings (ECG or central blood pressure/arterial stiffness values) will be performed for research purposes only and not for diagnostic purposes according to the methodology reported in Table S4. Once the ECG has been acquired in digital form, it will be stored on a password-protected computer in dedicated rooms of the two study centers. These data will be digitally transferred using an encrypted and password-protected system to the laboratory where the measurements envisaged by the project will be performed to determine the selected and aforementioned cardiovascular and renal instrumental biomarkers.

Furthermore, vascular augmentation index and central blood pressure will be characterized using multi-site peripheral pressure oscillometric methods as indicators of early vascular remodeling and damage, as also recommended by guidelines for estimating subclinical cardiovascular damage (Table 1). The main parameters obtained (Table S4) are characterized by high prognostic power in patients with varying degrees of cardiovascular risk, including those with high risk factors such as hypertension, diabetes, and chronic kidney disease.

##### Respiratory Disease Risk Biomarkers

The selected respiratory biomarkers were chosen for their ability to capture inflammatory, oxidative, and systemic stress responses associated with respiratory diseases and environmental exposure (Table 1 and Table 3). Reactive Oxygen Species (ROS), such as hydroxyl radical, hydroperoxyl radical, superoxide radical anion, excited-state singlet oxygen and ozone, play a key role in maintaining cellular redox balance and signaling. Their imbalance, referred to as oxidative stress, is frequently linked to exposure to environmental pollutants, including particulate matter, heavy metals, and airborne chemicals [110,111]. Elevated ROS levels can induce oxidative damage to lipids, proteins, and DNA, contributing to cellular dysfunction and chronic disease development. Moreover, ROS act as mediators in inflammatory pathways and can exacerbate the effects of toxic environmental exposures, making their detection and quantification in biological fluids essential for assessing individual susceptibility and the health impact of environmental stressors. Assessment of ROS generation may improve the understanding of individual susceptibility to pollutant-induced oxidative damage and respiratory disease risk [112]. Among analytical approaches, Electron Paramagnetic Resonance spectroscopy stands out as a unique technique capable of directly detecting spin-probe/trapped ROS adducts in biological systems, offering qualitative and quantitative information on radical identity and production rates that are otherwise difficult to capture with conventional indirect assays. In this study, Electron Paramagnetic Resonance methods with spin trapping or spin probes will be applied to measure ROS generation in biological fluids, cells and tissues, underlining their value for studies in redox biology and environmental health research [113,114,115].

Carbonic anhydrase 6, associated with inflammatory processes in the airways, may represent an early marker of alterations in the respiratory environment [116]. Carbonic anhydrase 6 is a secreted enzyme involved in maintaining acid-base balance and local pH homeostasis in the oral and respiratory mucosa. Altered expression or activity of carbonic anhydrase 6 has been linked to airway inflammation, oxidative stress, and changes in mucosal defense mechanisms, potentially preceding overt clinical symptoms. Finally, cortisol, which allows monitoring of the activation of the hypothalamic-pituitary-adrenal axis, is particularly relevant in subjects subjected to environmental stress [117,118]. As a key glucocorticoid hormone, cortisol regulates numerous physiological processes, including metabolism, immune response, and cardiovascular function. Its levels reflect both acute and chronic stress responses, making it a valuable biomarker for assessing the impact of environmental, psychological, or occupational stressors. Saliva, as a non-invasive approach, enables repeated sampling in real-life settings and is an easily accessible biofluid, offering a practical matrix for longitudinal studies of oxidative stress/inflammation, respiratory health, and hormonal profiling in population-based research. Moreover, saliva facilitates longitudinal monitoring and early detection of dysregulation in balance/unbalance redox responses, systemic inflammation, hormonal stress and hypothalamic-pituitary-adrenal axis responses, offering opportunities for preventive interventions. Integration of measure assessment with other biomarkers enhances the understanding of systemic responses to environmental challenges.

A single saliva sample will be collected from each subject (a non-invasive, convenient, easily repeatable, stress-free, minimal training, and well-tolerated approach). Using Salivette^®^ devices (SARSTEDT AG & Co. KG Sarstedtstraße 1, 51588 Nümbrecht, Germany) the saliva sample will be collected directly by the subject under the supervision of the nursing staff according to the specifications in Table S5. Samples will be treated according to the methods described in Table S5 until they are transferred to the specific analysis laboratory where they will be analyzed according to the methods reported in Table 3 and described in detail in Table S5.

##### Disease Risk Biomarkers and Biological Indicators of Aging

The analysis of disease risk biomarkers and biological indicators of aging will be based on the assessment of telomere length (TL) (Table 3), as an early indicator of cellular aging and risk of chronic degenerative diseases, and on the identification of epigenetic alterations through the assessment of the global methylation status of DNA extracted from whole blood. Therefore, TL was selected as an integrative biomarker of cumulative biological stress, disease susceptibility, and biological aging (Table 1). Telomeres are nucleoprotein structures composed of tandem TTAGGG DNA repeats and associated shelterin proteins, located at the ends of linear chromosomes, where they play a critical role in maintaining genomic stability and preventing chromosomal end-to-end fusions [119,120]. TL is a key determinant of cellular replicative capacity and overall cellular health. Indeed, telomeres progressively shorten with each cell division due to the end-replication problem, as well as through oxidative stress and inflammation [121]. Although telomere shortening is a natural consequence of chronological aging, accumulating evidence indicates that this process can be accelerated by environmental exposures, including air pollution, POPs, and other sources of oxidative stress [122,123]. Shortened TL has been associated with an increased risk of age-related diseases, including cardiovascular diseases, type 2 diabetes, and neurodegenerative disorders [124,125]. Conversely, the relationship between TL and cancer risk remains complex and not yet fully elucidated. While critically short telomeres can lead to genomic instability and potentially promote carcinogenesis, longer telomeres may also increase the proliferative potential of pre-malignant cells, thereby contributing to tumor development under certain conditions [126,127].

Telomere length will be interpreted as a marker of cumulative biological stress rather than as a disease-specific indicator. Analyses involving telomere length will account for major determinants such as age, sex, smoking habits, and available hematological parameters, to reduce confounding related to interindividual variability and leukocyte composition.

The epigenome constitutes a key interface in gene–environment interactions due to the dynamic and partially reversible nature of epigenetic modifications. This makes epigenetic marks valuable biomarkers of both prior environmental exposures and early biological effects, linking external stimuli to downstream molecular responses. Accordingly, epigenetic alterations are increasingly used as sensitive indicators of environmental stressors, including air pollution, heavy metals, and endocrine-disrupting chemicals [128,129].

Among epigenetic mechanisms, DNA methylation is the most widely investigated. It involves the addition of a methyl group (–CH_3_) to the 5′ position of cytosine residues, mainly at Cytosine-phosphate-Guanine dinucleotides, which are often enriched in Cytosine-phosphate-Guanine islands within gene promoter regions. This modification is generally associated with transcriptional repression, either by directly interfering with transcription factor binding or by recruiting proteins that induce chromatin compaction and gene silencing [130]. However, its functional impact depends on genomic context, as methylation within gene bodies may also be linked to active transcription [131].

DNA methylation patterns are responsive to environmental exposures and can reflect both short- and long-term adaptive processes. Pollutant exposure has been shown to induce global and locus-specific methylation changes, potentially mediated by oxidative stress, inflammation, and altered activity of DNA methyltransferases [132,133]. These alterations can modulate transcription factor binding and disrupt gene regulatory networks involved in key biological pathways such as detoxification, immune response, and cellular stress.

Given its central role in gene regulation, cellular differentiation, and tissue homeostasis, dysregulation of DNA methylation has been implicated in the development of several diseases, including cancer, cardiovascular conditions, metabolic disorders, and neurodegenerative diseases [134]. Therefore, DNA methylation profiling—using platforms such as the Illumina Infinium Methylation Screening Array—represents a powerful tool for investigating environmentally driven biological changes and identifying early biomarkers of disease susceptibility and biological aging (Table 1).

All samples will be treated according to the methods described in Table S6 until they are transferred to the specific analysis laboratory, where they will be analyzed according to the methods reported in Table 3 and described in detail in Table S6.

##### Metabolomic Profiling

Environmental pollutants that act as endocrine disruptors are associated with the development of metabolic diseases, including diabetes, obesity, and metabolic dysfunction-associated steatotic liver disease. Therefore, metabolomic profiling is essential to monitor any metabolic changes associated with exposure (Table 1) [135,136]. Metabolomic profiling includes measurements of lipid composition, amino acids, Krebs cycle intermediates, organic acids such as ketoacids, alpha- and beta-hydroxybutyrate, glycerate, and uric acid. All analyses will be performed in plasma, selected as the biological matrix to capture systemic metabolic alterations associated with environmental exposure. Analysis will be conducted using validated mass spectrometry-based platforms, with appropriate quality control procedures and internal standards to ensure analytical robustness and reproducibility. Innovative targeted and untargeted (global) metabolomic analyses will be performed to assess changes in the main metabolites involved in glucose, lipid, and amino acid metabolism, and associated with hepatic and systemic metabolic dysfunction, compared to control values [137]. Furthermore, measurement of tryptophan metabolites and glutathione precursors will allow monitoring the metabolic response to inflammation and any oxidative stress resulting from exposure [138], which can be correlated with salivary measurements.

All samples will be treated according to the methods described in Table S7 until they are transferred to the specific analysis laboratory where they will be analyzed according to the methods reported in Table 3 and described in detail in Table S7.

For metabolomic analyses, quality control samples will be included throughout the analytical sequence to monitor instrumental stability, analytical drift, and reproducibility. Data preprocessing will include peak detection, alignment, filtering of low-quality features, normalization, and, when necessary, batch-effect correction. Multivariable and univariable analyses will be conducted using appropriate statistical workflows, and multiple testing will be controlled using false discovery rate correction procedures.

##### Storage in the Biobank

For any further in-depth analysis, residual blood samples will be collected and stored in the Biobank at the Institute of Clinical Physiology for a period of 10 years, ensuring controlled access, continuous temperature monitoring and documented processing and custody chain, after which they will be destroyed.

The Biobank has been certified according to the ISO 9001:2015 standard [139] since 2020. The activities and security of the Institute of Clinical Physiology Biobank are defined by the Institutional Regulations (specific information available online at https://www.ifc.cnr.it/index.php/it/biologia-preclinica/biobanca (accessed on 13 May 2026)). Samples will be treated according to the methods described in Table S8.

#### 3.3.3. Application of the Study Population and Sampling Strategy

Considering (i) the availability of financial resources, (ii) the feasibility in terms of human resources, (iii) the study’s expected timeframe (December 2026), and (iv) the statistical power needed, 1000 subjects from the general population will be enrolled from the municipalities of Livorno, Collesalvetti (700 subjects), and Piombino (300 subjects) (Table 1). The decision on the sample size is due to (i) the demographic proportion between the two areas, (ii) the need to ensure adequate statistical power for stratified analyses, (iii) the greater environmental complexity and variability of exposures in the Livorno area, and (iv) operational feasibility. This distribution will allow an adequate representation of the different exposure contexts while ensuring analytical robustness (Table 1). Furthermore, this sample size will enable the identification, for the main heavy metals (As, Cd, Cr, Hg, Pb), for 1-hydroxypyrene and for the main VOCs, increases in urinary concentrations in the two NPCSs greater than 15% compared with available Italian reference values (http://www.sivr.it/, 14 May 2026; [140]) with a study power greater than 80%. This calculation is feasible only for the above-mentioned compounds because validated population reference values are currently available exclusively for them and not for the other substances.

The sample of 1000 subjects will be selected from the Regional Health Service, will be stratified by sex (males—M, females—F), age groups (20–34, 35–49, 50–64), and exposure areas based on the distance from the NPCSs’ centroid. The maximum distance of 10 km from the NPCSs’ centroids has been divided into four equal classes: class 1 (0–2.5 km), class 2 (2.5–5 km), class 3 (5–7.5 km), and class 4 (7.5–10 km). A distance-based approach was adopted to classify exposure because it better captures spatial gradients of environmental contamination than administrative boundaries. The identification of the maximum extension radius of the exposed area (10 km) is based on scientific literature. The distance-based classification was selected as a pragmatic and reproducible approach suitable for population-based recruitment and stratified sampling. We acknowledge that factors such as prevailing wind direction, emission sources, atmospheric dispersion patterns, local topography, and temporal variability in industrial activities may influence individual exposure levels. However, the integration of these parameters was not feasible within the present study design and available environmental datasets. Therefore, distance from the NPCS centroid was used as a proxy of potential environmental exposure, and this limitation will be considered when interpreting the findings (Table 1).

Georeferenced residential addresses will be used to assign each participant to the appropriate exposure category.

The sampling strategy is designed to ensure population representativeness across all combinations of sex, age classes, and exposure classes, thereby preserving comparability and analytical robustness in stratified analyses (Table 1).

To compensate for potential non-responders and ensure an adequate sample size within each stratification cell, an oversampling equal to six times the required number of subjects per cell is planned (Table S9).

#### 3.3.4. Inclusion and Exclusion Criteria for Recruitment

##### Inclusion Criteria

The inclusion criteria were defined to maximize the likelihood of capturing long-term environmental exposure while ensuring the feasibility and epidemiological robustness of the study. Eligible participants were adults aged 20–64 years residing in the study municipalities for at least five years. The selected age range reflects scientific, ethical, and operational considerations. Adults within this age group were expected to have more stable residential histories and long-term exposure profiles while reducing heterogeneity associated with developmental physiology in children and with multimorbidity, polypharmacy, and frailty in older adults, which could complicate the interpretation of biomarkers and health outcomes. Furthermore, limiting the study to the adult population facilitates a more direct assessment of environmental and occupational exposures, consistent with the primary objectives of the project. Children and older adults are recognized as particularly vulnerable populations; however, their inclusion would have required dedicated study protocols, age-specific biomarker interpretation, additional ethical procedures, and substantially greater clinical and logistical resources. These groups should therefore be considered priorities for future dedicated HBM investigations (Table 1).

Residence in the study municipalities and a minimum residence period of five years were required to increase the plausibility that the measured internal exposure reflected the environmental conditions of the investigated areas. The five-year criterion, consistent with previous HBM studies [141,142,143,144], was selected to ensure a stable pattern of chronic environmental exposure, considering the persistence and bioaccumulation potential of contaminants such as PFAS, heavy metals, PAHs, and PCBs. This time window also reduces exposure misclassification related to previous residence in other areas, thereby improving the epidemiological reliability of the study (Table 1).

##### Exclusion Criteria

The exclusion criteria adopted are those commonly used in HBM studies involving generally healthy populations and aim to avoid clinical conditions that could (i) interfere with the correct interpretation of environmental biomarkers, (ii) pose additional risks to participants and (iii) influence health status independently of environmental exposure.

Therefore, individuals with active chronic infectious diseases, pregnancy, specific allergies that prevent sample collection procedures, or an inability to provide valid informed consent will be excluded (Table 1).

#### 3.3.5. Recruiting and Enrolment

##### Contact and Recruitment Procedure

The recruitment process will involve two telephone contacts. During the first call, operators will briefly describe the study, verify eligibility criteria, and collect the subject’s willingness to participate. Individuals who express interest will be contacted again in a second call, during which an appointment will be scheduled at the study centers for the planned procedures (Table 1).

Telephone-based recruitment may introduce selection bias by reducing participation among hard-to-reach individuals. However, the target population of this study consists of community-dwelling adults aged 20–64 years. Children, institutionalized elderly people, and hospitalized individuals are not part of the predefined study population. This potential selection issue will be acknowledged when interpreting the representativeness of the sample enrolled (Table 1).

##### Enrolment Visit

On the day of the appointment, participants will be welcomed at the designated center, where a standardized medical history assessment will be conducted to confirm eligibility for enrolment. Eligible subjects will then be asked to sign the informed consent form, which will be recorded using a Research Electronic Data Capture (REDCap) electronic Case Report Form (eCRF) (Table 1, Figure 1). More details about the use of the REDCap eCRF are provided in Section 3.3.6.

Immediately afterward, biological samples (blood, urine, and saliva) will be collected. Subjects will be asked to fast for at least eight hours, to arrive at the study centers without having drunk anything other than water and without brushing their teeth, and to donate urine from the second void. Two instrumental examinations will then be carried out: a resting ECG and a standardized blood pressure measurement. Finally, participants will complete an online questionnaire using a tablet provided by the center or their personal device, with trained staff available for assistance if needed. The questionnaire will collect information on medical history, lifestyle, environmental and occupational exposures, and sociodemographic characteristics.

Figure 1 illustrates the entire procedure.

#### 3.3.6. REDCap eCase Report Form (REDCap eCRF)

Data collection will be carried out using a customized eCRF specifically developed for the project. Although spreadsheet software such as Excel or Access may support local data management activities, they do not provide the security, audit trail, role-based access control and regulatory compliance required for multicentre clinical research. Data will be collected and managed using REDCap tools (REDCap 15.5.35) hosted on the REDCap platform at the CNR-IFC Institute. REDCap platform will be used for this study in compliance with good clinical practices, current legislation on data protection (General Data Protection Regulation—GDPR), and data quality assessed through Accuracy, Completeness, Consistency, Integrity, and Timing criteria [145,146,147]. Electronic data management will be performed using REDCap, a secure, web-based platform fully compliant with GDPR, designed to support data capture for research studies and widely used in clinical and epidemiological research (Table 1). REDCap enables real-time data entry and provides advanced functionalities such as automated field validation, a complete audit trail, regular backups, and compliance with major data protection standards [148]. A key feature of the system is its access control architecture, which allows each team member to be assigned personalized credentials with role-based authorization levels (e.g., data entry, monitoring, data management, scientific oversight), ensuring that users can access only the functions and datasets relevant to their responsibilities (Table 1). The eCRF has a modular structure integrating sociodemographic, medical history, clinical, lifestyle, environmental and occupational exposure information, instrumental measurements, and laboratory analytical results. It is designed to ensure uniform data entry and minimize input errors through automatic consistency checks, mandatory fields, and predefined menus.

#### 3.3.7. Questionnaire

To collect relevant information, each subject will be administered a questionnaire, divided into eight sections and approximately 80 questions, as follows:

Section A—Personal Data (14 questions)
Section B—Sports Activities (3 questions)
Section C—Smoking and Alcohol Consumption (4 questions)
Section D—Medical history and clinical conditions (15 questions)
Section E—Environmental and socioeconomic conditions (13 questions)
Section F—Exposure to chemical and physical agents (2 questions)
Section G—Eating habits and diet (14 questions)
Section H—Perception of dangers and risks (14 questions).

The questionnaire is not validated but is similar to those used in previous environmental and health-related investigations (CISAS Project—Center for Advanced Studies on the Environment and Impacts on the Ecosystem and Human Health and SEpiAs—Epidemiological Surveillance in Areas Affected by Natural or Man-Made Arsenic Pollution; CCM SEpiAs—Studies on Exposure and Early-Effect Markers in Areas with Arsenic Pollution: Methods and Results of the SEpiAs Project) (Table 1).

To avoid compilation errors, the questionnaires will be administered online with the assistance of a healthcare professional in case further information is needed.

#### 3.3.8. Epidemiologic and Statistical Analysis

Measurements below the limit of detection (LOD) or limit of quantification (LOQ) will be handled according to the proportion of censored values for each analyte. For biomarkers with a low proportion of values below the LOD, substitution methods such as LOD/√2 may be applied. For analytes with a higher proportion of censored observations, statistical methods appropriate for left-censored environmental data will be considered. Analytes with very low detection frequencies will not be interpreted as continuous exposure indicators and may be described only in terms of detection frequency. Analytical uncertainty and potential statistical noise at very low concentration ranges will be considered when interpreting associations (Table 1).

Missing data patterns will be assessed, and multiple imputation using predictive mean matching will be applied when appropriate to minimize potential bias and loss of statistical power.

Descriptive statistics will be used to summarize the distribution of biomarkers using percentiles, arithmetic means, geometric means, standard deviations, and to identify outliers according to the Italian Society of Reference Values and the Italian National Institute of Health, and to the literature reference values observed in other investigations and published in accredited scientific journals or institutional reports, with the aim of placing the observed biomarker concentrations within the national reference context of populations/communities living with different risk characteristics (Table 1).

The integration of anthropometric, biochemical and clinical data will allow the calculation of validated multiparametric score indicators of cardiovascular, renal, metabolic, and hepatic risk.

Associations between exposure biomarkers, biological response markers, health indicators, and composite risk scores will be assessed using multivariable regression models. Exposure biomarkers and environmental, occupational, demographic, socioeconomic, behavioral, dietary, and clinical variables collected through questionnaires will be considered as potential determinants of biological responses and health-risk indicators. Association models will be adjusted for relevant confounding factors identified a priori based on biological plausibility and existing scientific evidence, including age, sex, body mass index, smoking status, alcohol consumption, educational level, occupational exposures, dietary habits, physical activity, medication use, pre-existing health conditions, etc. Regression diagnostics will be performed to assess model assumptions, including linearity, normality of residuals, homoscedasticity, and multicollinearity. When necessary, biomarker concentrations will be transformed to improve model fit and satisfy statistical assumptions. Results will be expressed as regression coefficients and corresponding 95% confidence intervals, providing estimates of the magnitude and direction of associations between environmental exposures and biological or clinical outcomes.

To investigate the complex relationships linking environmental exposures, biological responses, and early health effects, integrative analytical approaches will be applied. Beyond conventional regression analyses, the study will explore patterns of association among environmental exposures, biological responses, and health-related indicators through integrated exposome-oriented approaches. Multilevel analytical strategies, including mediation analyses, structural equation modeling, network analyses, and machine-learning techniques, will be employed to evaluate relationships among exposure biomarkers, intermediate biological processes (e.g., oxidative stress, inflammation, metabolic alterations), molecular markers of biological aging and susceptibility, such as telomere length and DNA methylation, and downstream indicators of organ dysfunction or disease risk. These approaches are intended to identify potential exposure–response patterns, characterize complex exposure mixtures, and generate hypotheses regarding biological mechanisms that may underlie observed associations. In addition, predictive modeling techniques, including supervised machine-learning algorithms, will be used to identify exposure profiles, rank the relative importance of biomarkers, and derive preliminary predictive estimates of early biological and clinical alterations associated with environmental exposures. The resulting findings should be interpreted as exploratory and hypothesis-generating and may help inform future longitudinal epidemiological studies with repeated measurements over time aimed at evaluating temporal relationships and potential causal pathways.

The integration of conventional epidemiological analyses with advanced multilevel modeling approaches may improve the identification of complex exposure–response patterns linking environmental contaminants, intermediate biological mechanisms, and early health effects.

### 3.4. Ethics and Dissemination in the INSINERGIA_RT Study

The INSINERGIA_RT protocol for the Tuscany region was approved by the Tuscany Region Ethics Committee—Northwest Area (approval number 29144_BARBIERI, 4 September 2025). The study will be conducted considering regulatory requirements and legal compliance. Furthermore, any intervention will comply with the ethical principles established in the Declaration of Helsinki and its revisions. Prior to enrolment, all subjects will receive comprehensive information about the study. Data will be processed in accordance with the provisions of Legislative Decree No. 679/2016 of 30 June 2003, as amended, the GDPR, and Resolution No. 52 of 24 July 2008, as amended, as well as Legislative Decree No. 101 of 10 August 2018, “Provisions for the adaptation of national legislation to the provisions of the GDPR.” Specifically, the processing of personal data (both common personal data and data belonging to the specific category of data such as health data) for project activities is carried out for the purposes of scientific research and statistical analysis on the basis of the data subject’s consent pursuant to the provisions of Article 13 of the GDPR. 6, paragraph 1, letter (a) of the GDPR for ordinary personal data and art. 9, paragraph 2, letter (a) of the GDPR for sensitive data.

To identify the subjects throughout the study, a pseudonymization procedure will be implemented by assigning, to each enrolled subject, a unique alphanumeric identification code (ID) containing no information that could be linked to the subject’s identity. This ID will be used to label the corresponding blood, urine, and saliva collection tubes and identify the corresponding ECG and BP+ readings. A master file, accessible only by the principal investigator, will be created containing information for each subject, such as name, surname, date of birth, and the corresponding ID. All anamnestic, anthropometric, clinical, biochemical, instrumental, and questionnaire data will be manually entered into an online eCRF (as detailed in Section 3.3.6), in accordance with GDPR regulations, not containing data that could be used to trace the subjects’ identity. Operators will be able to access the eCRF for completion and viewing at different security and privacy levels depending on their role. The final dataset for epidemiological analyses will not contain sensitive information that could be used to identify participants but only pseudonymized data.

All results will be disseminated exclusively in anonymized and aggregated data form through scientific publications, conferences, institutional reports, and public communication activities. The research data will be published in aggregated form, guaranteeing participant anonymity, via open-source platforms on the internet, social networks, and at scientific events such as conferences and congresses. Furthermore, scientific publications will be produced to share the results obtained with the scientific community and stakeholders. Publications will be produced both during and after the project.

A continuous ethical monitoring plan will ensure adherence to the protocol, data quality procedures, and participant protection. Amendments will be submitted to the Ethics Committee.

Risks associated with participation are minimal, while participants may benefit from receiving clinically relevant information (e.g., blood pressure, ECG).

A structured plan for returning results—both at the individual level (where clinically appropriate) and at the community level (reports, public meetings)—will ensure transparency.

The dissemination strategy will follow FAIR data principles (Findable, Accessible, Interoperable, Reusable) for aggregated datasets.

The project will also include actions of community and stakeholder engagement, ensuring that results inform local health authorities, citizens, and environmental agencies.

## 4. Discussion

The present study contributes to advancing HBM by introducing the descriptive use of biomarkers and proposing a transparent and structured framework for their selection and integration in complex environmental settings.

Rather than reviewing contaminants and associated health effects, this study addresses a critical gap in the literature: the lack of structured and reproducible approaches for designing HBM studies in complex contaminated settings.

### 4.1. From Biomarker Lists to Structured Decision-Support Process

Most HBM studies include biomarkers of exposure and effect but provide limited information on the rationale guiding their selection. As a result, methodological decisions often remain implicit, limiting reproducibility, comparability, and methodological transparency.

In contrast, this approach explicitly documents the rationale behind site selection, sampling strategy, contaminant prioritization, and biomarker selection, including the supporting evidence, alternatives considered, and operational constraints. An important contribution of this work is not the identification of novel methodological criteria, but the explicit documentation of how commonly recognized HBM design principles—including biological plausibility, epidemiological relevance, analytical feasibility, temporal stability, participant burden, and resource constraints—were operationalized to support concrete methodological decisions within a real-world contaminated-site study. So, this work provides a practical methodological reference for the design of HBM studies in contaminated sites.

### 4.2. Mechanistic Integration as a Core Innovation

A key innovation of this study is the adoption of a mechanistic, multi-level approach to biomarker selection. Rather than relying on individual biomarkers, the proposed panel captures interconnected biological pathways linking environmental exposure to early biological and health effects. In particular, the integration of oxidative stress and inflammation as upstream pathways provides a biologically coherent basis for interpreting heterogeneous exposures, as these mechanisms are shared across multiple classes of contaminants.

This upstream layer is complemented by downstream indicators of subclinical organ damage, including cardiovascular and renal markers, and by higher-level integrative biomarkers such as telomere length, epigenetic modifications, and metabolomic profiles. This hierarchical structure captures biological responses from early molecular changes to systemic effects.

An additional strength of this approach is its improved biological interpretability, as exposure-biomarker associations can be evaluated within a coherent mechanistic framework.

Importantly, the biomarker panel was tailored to the environmental contamination profile, epidemiological evidence, and objectives of the investigated NPCSs. Different exposure scenarios may legitimately require different biomarker panels while following the same methodological reasoning.

### 4.3. Balancing Biological Relevance and Feasibility

Another important contribution of this study is the explicit consideration of the balance between biological relevance and operational feasibility.

Biomarker selection was based not only on scientific relevance, but also on:

- analytical robustness and standardization;
- feasibility in field conditions;
- acceptability for participants;
- cost and logistical constraints.

These elements are particularly critical in population-based studies in contaminated sites, where large sample sizes and complex field conditions may limit the applicability of highly specialized or invasive measurements.

By explicitly incorporating these constraints into the study design, the proposed framework offers a realistic and reproducible approach to HBM research in contaminated sites.

### 4.4. Temporal Variability and Biomarkers Interpretation

Temporal variability is one of the major methodological challenges in HBM studies and should be considered during both study design and result interpretation. Preference may be given to biomarkers reflecting relatively stable biological processes, while sampling and pre-analytical procedures should be standardized to minimize variability unrelated to environmental exposures.

At the same time, the potential for substantial intra-individual variability should be acknowledged for certain biomarkers, particularly those related to oxidative stress, inflammation, and metabolomic profiles. Depending on the study objectives, repeated sampling designs may be considered to better characterize temporal fluctuations and improve exposure assessment.

The choice between single and repeated sampling strategies should balance scientific objectives, biomarker characteristics, participant burden, logistical feasibility, and available resources. The implications of temporal variability should be explicitly considered when interpreting exposure–response relationships and early indicators of biological and health effects.

In this study, temporal variability was addressed through several strategies.

First, preference was given to biomarkers that reflect relatively stable biological processes (e.g., systemic inflammation, telomere length), reducing susceptibility to short-term fluctuations. Second, sampling procedures were standardized as much as possible to minimize pre-analytical variability.

Nevertheless, it is important to acknowledge that some biomarkers—particularly those related to oxidative stress and metabolomics—may exhibit significant intra-individual variability. This represents an inherent limitation of cross-sectional HBM studies and should be considered when interpreting the results.

Future longitudinal studies with repeated measurements could better characterize temporal dynamics and causal relationships.

### 4.5. Implications for Study Design in Contaminated Sites

The methodological approach presented here may support the design of future HBM studies conducted in complex contaminated environments.

By clearly articulating the criteria used for:

- selecting study sites;
- defining the target population;
- prioritizing contaminants;
- choosing biomarker panels.

The main value of this approach is the explicit documentation of methodological choices, improving transparency and facilitating adaptation to different environmental contexts. By integrating environmental, toxicological, and epidemiological evidence within a coherent methodological process, the study provides a practical methodological reference for researchers facing similar design challenges while supporting a more biologically informed interpretation of health risks in contaminated areas.

#### 4.5.1. Integration of Feasibility Constraints

A key component of any HBM framework is the explicit integration of operational and feasibility considerations into study design. Methodological choices should be evaluated not only according to scientific relevance but also in relation to available resources, laboratory capacity and standardization, participant burden, data management requirements, and logistical feasibility.

Balancing scientific rigor with operational feasibility is essential for the successful implementation of population-based HBM studies. Therefore, decisions regarding study design, biomarker selection, analytical methods, sampling strategies, and quality assurance procedures should consider both methodological robustness and practical feasibility.

This approach supports the development of scientifically sound and operationally sustainable studies capable of generating meaningful information on environmental exposures and their potential biological and health impacts.

#### 4.5.2. Framework Transferability

The methodological approach described in this study may facilitate the planning of HBM investigations in other complex contaminated settings. By explicitly documenting the criteria, assumptions, alternative options, and trade-offs underlying methodological decisions, this work illustrates how multidisciplinary evidence can be integrated into study design.

Although the methodological process was developed for the INSINERGIA_RT study, the underlying decision criteria—including the integration of environmental evidence, epidemiological information, toxicological knowledge, analytical feasibility, and operational constraints—may also be applicable to other contaminated-site HBM studies. However, the specific methodological choices should always be adapted to the characteristics of the investigated population, contaminants of concern, study objectives, and available resources.

This framework is not intended as a universally applicable model but as a flexible approach that can be adapted to different exposure scenarios, study objectives, and resource constraints. Rather, it documents the methodological reasoning that guided the design of a complex population-based HBM study before the availability of study results. The practical value of this decision process will ultimately be evaluated through the implementation of the INSINERGIA_RT study and future applications in other contaminated-site settings. Overall, the explicit documentation of methodological choices enhances transparency, supports multidisciplinary integration, and may improve the reproducibility of future HBM studies.

### 4.6. Methodological Relevance for Future HBM Studies

More transparent and standardized approaches to biomarker selection may improve cross-study comparability, facilitate the development of reference values, and strengthen evidence-based policy decision-support.

In this context, the integration of biomarkers of effect—particularly those linked to early biological alterations—may strengthen the capacity of HBM to inform preventive strategies, enabling earlier identification of at-risk populations and more targeted public health interventions.

### 4.7. Limitations

Several limitations should be considered. First, the selection of biomarkers inevitably reflects a balance between scientific ambition and feasibility, and some potentially informative markers may not have been included due to practical constraints. Second, cross-sectional design limits the ability to infer temporal relationships between exposure and biological response. Furthermore, variability in biomarker measurements, particularly for dynamic molecular endpoints, may introduce uncertainty that should be considered in interpretation. Finally, the restriction to adults aged 20–64 years represents a limitation in terms of population representativeness. Children and older adults are recognized as particularly vulnerable populations, but their inclusion would have required dedicated protocols, age-specific biomarker interpretation and additional ethical and logistical procedures.

Despite these limitations, a major strength of this work is the transparent documentation of the methodological rationale underlying all key study design decisions.

Because the INSINERGIA_RT study is currently ongoing, the effectiveness of the methodological decisions described here cannot yet be evaluated through study outcomes. Nevertheless, documenting the reasoning underlying these decisions before data analysis increases transparency and may facilitate methodological consistency across future HBM investigations.

## 5. Conclusions

This manuscript describes the methodological process used to design the INSINERGIA_RT human biomonitoring study and illustrates how environmental, toxicological, epidemiological, analytical, statistical, and operational considerations were integrated into study planning. The integration of detailed exposure assessment, a wide panel of biomarkers capturing multiple biological pathways, standardized data collection procedures, and advanced analytical methods represents a key strength of this approach. This multidimensional structure allows not only the quantification of internal exposure, but also the identification of early biological effects and potential mechanisms linking environmental contamination to health outcomes. These methodological choices resulted from a structured evaluation of scientific evidence, epidemiological priorities, analytical feasibility, and operational constraints.

A major strength of the proposed protocol is its harmonized, comprehensive, and scalable structure, which integrates environmental, clinical, molecular, and behavioral information within a single methodological framework. This approach enables consistent comparisons across different study areas and populations, supports the identification of complex exposure patterns, and strengthens the interpretation of exposure–effect relationships. The inclusion of biological aging indicators, metabolomic profiling, and mechanistically informed biomarkers extends the scope of traditional HBM approaches and may improve the identification of early biological alterations with environmental exposures.

Its application in different settings may facilitate the identification of vulnerable populations, the evaluation of exposure–response relationships, and the prioritization of public health interventions and remediation strategies.

The methodological approach described in this work may strengthen environmental health surveillance systems by improving transparency in study design and supporting evidence-based environmental health research.

In conclusion, this study contributes to the HBM literature by documenting the methodological rationale underlying the design of a population-based HBM study in two NPCSs. Its novelty lies not only in the selected biomarker panel but also in the explicit integration of environmental, toxicological, epidemiological, analytical, statistical, and operational considerations within a coherent study design framework. The practical value of this approach will ultimately be assessed through the implementation of the INSINERGIA_RT study. Nevertheless, by making methodological reasoning explicit, this work provides a useful methodological reference for researchers designing HBM studies in comparable contaminated-site settings and may facilitate future longitudinal investigations.

## Figures and Tables

**Table 2:** Hierarchical classification of study endpoints according to the proposed mechanistic multi-level framework.

Level	Endpoints	Purpose
Primary endpoints	Exposure biomarkers	Assessment of internal dose and characterization of environmental exposure
Secondary endpoints	Oxidative stress and inflammation biomarkers, cardiovascular, renal and respiratory biomarkers	Identification of early biological responses and subclinical target-organ effects
Exploratory endpoints	Metabolomics, telomere length, DNA methylation, other omics-based biomarkers	Evaluation of cumulative biological effects and identification of novel biological multi-level pathways associated with environmental exposures

**Table 3:** Application of the structured decision-support process to biomarker selection.

Decision-Making Considerations	Final Selection	Why This Choice?
1. Exposure biomarkers		
Environmental monitoring data; toxicological relevance; persistence; expected exposure in Livorno and Piombino	PFAS, NDL-PCBs, DL-PCBs, PCDDs/PCDFs, OCPs, OPPs, VOCs, PAHs, heavy metals. Mercury and Arsenic	Highest environmental relevance for the investigated NPCSs
2. Chemical and biological analytes		
Association with the expected exposure; reliability in providing accurate information about the subjects’ health risk profile	More than 60 analytes (see Supplementary Material)	Ability to characterize metabolic, hepatic, renal and cardiovascular function; support the interpretation of exposure and effect biomarkers;
3. Cardiovascular biomarkers		
SENTIERI evidence; biological plausibility; inflammatory pathways; analytical robustness	Inflammatory status (high-sensitivity C-reactive protein; interleukin 6, 1β, 18, tumor necrosis factor, and monocyte chemoattractant protein-1)	Central role as key mediators and integrators of systemic inflammatory responses
SENTIERI evidence; biological plausibility; inflammatory pathways; analytical robustness	Oxidative stress biomarkers (F2 isoprostanes, 3-nitrotyrosine, thiobarbituric acid reactive substances, 4-hydroxynonenal)	Ability to reflect oxidative damage to lipids and proteins, a key mechanistic pathway linking environmental contaminants to disease
Non-invasive biomedical devices	Cardiac age, Pulse Wave Analysis, central Blood Pressure, Augmentation index, Cardiac Output, Total Peripheral vascular Resistance	Capacity to measure well-known indicators of subclinical damage as a result of exposure to cardiovascular risk factors
4. Renal impairment biomarkers		
PFAS nephrotoxicity; metals; analytical validation	Renal impairment (cystatin C, beta-2 microglobulin, albuminuria, creatininuria and their ratio, kidney injury molecule 1, neutrophil gelatinase-associated lipocalin, beta-2 microglobulin)	Well-established nephrotoxic potential of several contaminants of concern
5. Respiratory biomarkers		
SENTIERI respiratory findings; mechanistic plausibility	Respiratory risk biomarkers (Reactive Oxygen Species, 8-hydroxydeoxyguanosine for oxidative DNA damage, cortisol concentration, Carbonic anhydrase 6)	Pathophysiological relevance and ability to reflect alterations in respiratory disease mechanisms
6. Disease risk biomarkers and biological indicators of aging		
Early biological aging; feasibility	Telomere length	Indicator of cumulative biological stress
Early biological aging; feasibility	DNA methylation	Sensitive indicators of environmental stressors
7. Metabolomic profiling		
Mechanistic interpretation; discovery potential	Fatty acids, amino acids, Krebs cycle intermediates, organic acids and ketoacids, alpha- and beta-hydroxybutyrate, glycerate, and uric acid, etc.	Comprehensive characterization of metabolic perturbations associated with exposure

Legend—NDL-PCBs: Non-Dioxin-Like Polychlorobiphenyls; DL-PCBs: Dioxin-Like Polychlorobiphenyls; OCPs: OrganoChlorine Pesticides; OPPs: OrganoPhosphorus Pesticides; PCDDs/PCDFs: PolyChlorinated Dibenzo-p-Dioxins and DibenzoFurans; PFAS: PerFluoroAlkyl Substances; VOCs: Volatile Organic Compounds; PAHs: Polycyclic Aromatic Hydrocarbons; NPCSs: National Contaminated Priority Sites.

## Data Availability

This manuscript describes a study protocol. No new data were created or analyzed in this study. Data sharing is not applicable to this article.

## Supplementary Materials

The following supporting information can be downloaded at: https://www.mdpi.com/article/10.3390/toxics14070616/s1, Figure S1: Delimitation of the National Priority Contaminated Site (NPCS) (in yellow) of Livorno. Source https://bonifichesiticontaminati.mite.gov.it/sin-36/ (accessed 15 May 2026); Figure S2: Delimitation of the National Priority Contaminated Site (NPCS) (in yellow) of Piombino. Source https://bonifichesiticontaminati.mite.gov.it/sin-9/ (accessed on 15 May 2026); Table S1: Exposure biomarkers identified, treatment and analysis methodology for blood samples; Table S2: Exposure biomarkers identified, treatment and analysis methodology for urine samples; Table S3: Selected analytes and respective methodologies for clinical biochemistry analysis of blood samples, the description, the tube type, and the storage method are also provided; Table S4: Cardiovascular and renal risk biomarkers identified, treatment and analysis methodology for blood and urine samples. Characteristics of instrumental measurements are also reported; Table S5: Respiratory disease risk biomarkers identified, treatment and methodology for saliva samples; Table S6: Disease risk biomarkers and biological aging indicators identified, treatment and analysis methodology for blood samples; Table S7: Treatment and analysis methodology for blood samples for metabolomic profiling; Table S8: Procedure for collecting blood samples to be stored in the biobank; Table S9: Distribution by municipality, sex, age group and exposure of the subjects to be recruited and the subjects extracted from the Registry of Assisted Persons.

## Abbreviations

The following abbreviations are used in this manuscript:

ECG	ElectroCardioGram
eCRF	electronic Case Report Form
GDPR	General Data Protection Regulation
HBM	Human BioMonitoring
INSINERGIA_RT	INISNERGIA_RT—INSINERGIA Tuscany Region
NPCS	National Priority Contaminated Sites
PAHs	Polyciclic Aromatic Hydrocarbons
PCBs	PolyChlorinated Biphenyls
PFAS	Per- and Polyfluoroalkyl Substances
POPs	Persistent Organic Pollutants
REDCap	Research Electronic Data Capture
ROS	Reactive Oxygen Species
SENTIERI	Studio Epidemiologico Nazionale dei Territori e Insediamenti Esposti a Rischio di Inquinamento [Nationale epidemiological studies of territories and settlements exposed to pollution risk]
VOCs	Volatile Organic Compounds
TL	Telomere Length
