# Supplementary material for: From Environmental Evidence to Biomarker Selection: A Structured Decision-Support Process for Human Biomonitoring Studies in Contaminated Sites
Source: Toxics. 2026 Jul 15;14(7):616. doi: 10.3390/toxics14070616 (PMC13416600; doi:10.3390/toxics14070616)
Supplement: Supplementary file 1 [file toxics-14-00616-s001.zip › toxics-4349640-supplementary.pdf]

Supplementary Material - Application of the Decision-Making Framework in the two Tuscan NPCSs

S1. The National Priority Contamination Site (NPCS) of Livorno

The Livorno NPCS (initially defined with Ministerial Decree of 24 February 2003 and definitively re-defined with the Decree of the Minister for Ecological Transition of 17 November 2021) is located within the two municipalities of Livorno and Collesalvetti (169.336 total inhabitants on 1st January 2025, https://demo.istat.it/app/?i=POS&l=it) and covers a total area of approximately 206 hectares, and does not include any coastal areas (Figure S1).

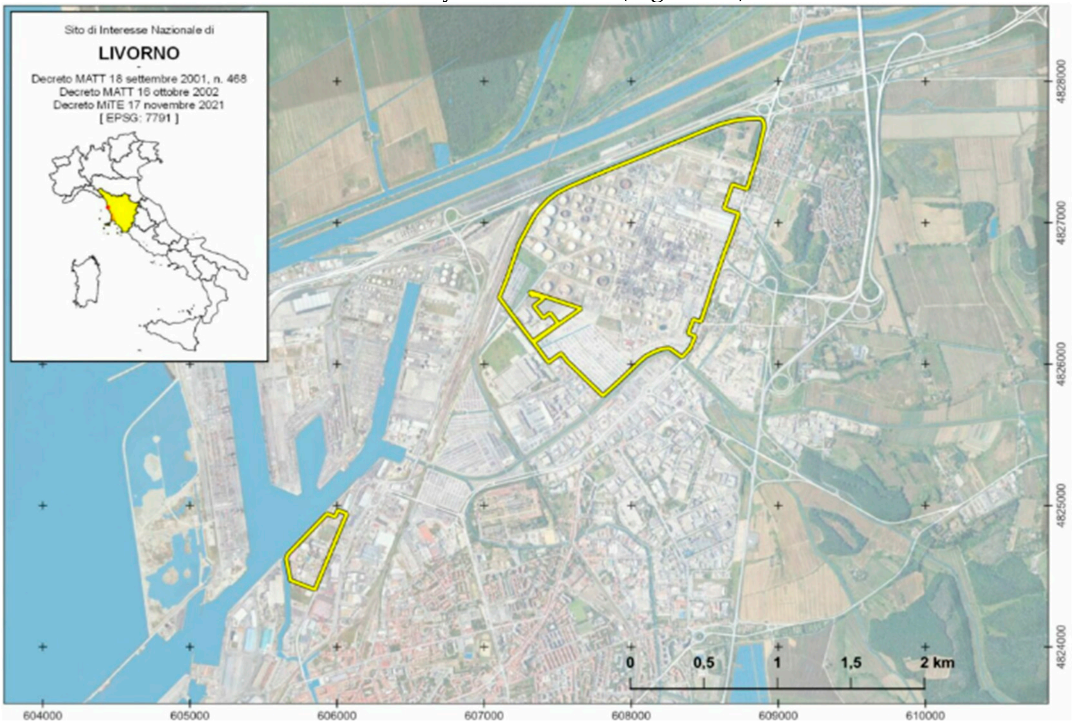

Figure S1. Delimitation of the National Priority Contaminated Site (NPCS) (in yellow) of Livorno. Source https://bonifichesiticontaminati.mite.gov.it/sin-36/ (accessed on 15 May 2026)

Within the Livorno NPCS, industrial and commercial activities include refinery-related areas, coastal storage facilities, artisanal–industrial activities, and port areas. The Livorno refinery, located between Livorno and Collesalvetti, covers approximately 182 hectares and includes a thermoelectric plant and other industrial facilities.

Site characterization studies have identified widespread soil contamination exceeding threshold levels for metals (e.g., arsenic, chromium, cadmium, mercury, nickel, lead, and zinc), hydrocarbons, methyl tert-butyl ether, and polycyclic aromatic hydrocarbons. Groundwater contamination is mainly associated with hydrocarbons and aromatic compounds, while in other industrial areas, including the former ENEL power plant, contamination also involves chlorinated solvents. Additional groundwater pollution sources have been detected in surrounding areas.

Epidemiological evidence from the VI SENTIERI Report highlights health impacts consistent with long-term environmental exposure in the Livorno NPCS

[8]. Increased overall mortality and excesses of malignant tumors, particularly lung cancer and pleural mesothelioma, as well as cardiovascular and digestive diseases, have been reported. Elevated risks of mesothelioma-related hospitalizations and congenital anomalies further support the relevance of environmental exposure in this area [8].

S2. The National Priority Contaminated Site (NPCS) of Piombino

The Piombino NPCS (initially defined with Ministerial Decree of 10 January 2000 and subsequently expanded by decree of 7 April 2006) falls within the municipality of Piombino (32.364 inhabitants on 1st January 2025, https://demo.istat.it/app/?i=POS&l=it). The Piombino NPCS primarily includes a large industrial hub, the Piombino port area, the ENEL Torre del Sale thermoelectric power plant, the adjacent marine area, and the "Poggio ai Venti" waste dump, for a total area of approximately 945 hectares on land; the marine area in front of the site, running parallel to the coast, extends approximately 3 km offshore (Figure S2).

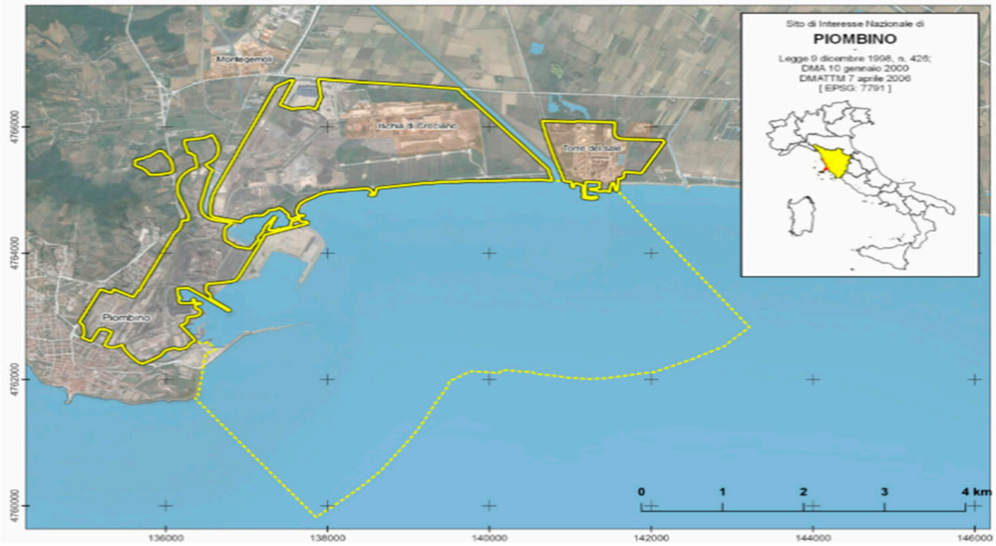

Figure S2. Delimitation of the National Priority Contaminated Site (NPCS) (in yellow) of Piombino. Source https://bonifichesiticontaminati.mite.gov.it/sin-9/ (accessed on 15 May 2026).

Until 2014, the main industrial activity in the Piombino NPCS was steelmaking, covering approximately 560 hectares, while current operations are limited to rolling processes for rails, profiles, and pipe coating. Additional areas include unused industrial land and public spaces along the northern shoreline. The Torre del Sale thermoelectric power plant has been inactive since 2014, and two decommissioned municipal waste landfills are located near the northern boundary of the site.

Environmental characterization studies have revealed contamination of both soil and groundwater. Soil contamination is mainly associated with arsenic, total chromium, vanadium, zinc, hydrocarbons (C>12), and polycyclic aromatic hydrocarbons, while cadmium, nickel, lead, mercury, copper, benzene, and chloromethane are present at lower concentrations. Groundwater contamination is characterized by elevated levels of metals (particularly arsenic, iron, and manganese), benzene, and organochlorine solvents, with additional contributions from lead, hexavalent chromium, nickel, PCBs, PAHs, and hydrocarbons.

These contamination patterns are linked to long-standing industrial activities, including fertilizer production and sulfuric acid processing, which historically generated waste materials such as pyrite ash.

Epidemiological evidence from the VI SENTIERI Report indicates increased overall mortality in the Piombino NPCS, with excesses in bladder cancer (both sexes), lung and pleural cancers (men), and cardiovascular and digestive diseases [8]. Additional increases are reported for hospitalizations due to malignant tumors and digestive diseases, as well as congenital anomalies, supporting the impact of long-term environmental exposure [8].

S3. Health effects of the main pollutants in the NPCSs

The classes of pollutants considered in the framework of the INSINERGIA_RT study are POPs: among them dioxins, PCBs, OCPs and OPPs, PAHs, VOCs, PFASs, metals and metalloids such as As and Hg.

The main health effects induced by these substances are briefly reported below.

S3.1. Persistent Organic Pollutants (POPs)

Dioxins - The term "dioxins" refers to a family of 210 polychlorinated aromatic chemical compounds divided into the classes PolyChlorinated DibenzoDioxins (PCDDs), composed of 75 congeners, and PolyChlorinated DibenzoFurans (PCDFs), composed of 135 congeners, distinguished by their remarkable environmental persistence, tendency to bioaccumulate, and considerable toxic potential [9,10]. Of these congeners, 17 are considered toxicologically relevant, the most toxic of which is 2,3,7,8-tetrachlorodibenzo-p-dioxin. Toxicity is expressed as Toxic Equivalent relative to 2,3,7,8-tetrachlorodibenzo-p-dioxin, which is classified as a Group 1 carcinogen by the International Agency for Research on Cancer. PCDDs and PCDFs are part of the POPs, the so-called "dirty dozen," together with PCBs, hexachlorobenzene, and the pesticides aldrin, chlordane, DDT, dieldrin, endrin, heptachlor, mirex, and toxaphene. Epidemiological studies have linked high exposures to increased risk of all cancers combined, as well as specific types like soft-tissue sarcoma and non-Hodgkin lymphoma [11-13], to interfere with multiple endocrine systems (thyroid, steroid hormone, and insulin signalling pathways [14] contributing to reproductive problems, metabolic disorders (diabetes, obesity) [15], to cognitive and behavioral deficits in children, as well as impairments in motor function [16], to chloracne [17]. Additionally, dioxins have been associated with cardiovascular and liver function disturbances [18].

PolyChlorinated Biphenyls (PCBs) - PCBs are artificial aromatic organic compounds present in the environment in mixtures. They contain 209 different substances, called congeners, exhibiting endocrine-disrupting properties. Of the 209 congeners, 12 have chemical-physical characteristics and toxicological properties comparable to 2,3,7,8-tetrachlorodibenzodioxin, the most toxic congener of the dioxin family, and are called dioxin-like or dioxin-like PCBs (DL-PCBs) [19]. The remainder are called non-dioxin-like PCBs (NDL-PCBs). PCBs are a group of POPs: toxic, synthetic chemicals characterized by bioaccumulation, ubiquity, and persistence. Numerous epidemiological studies associate exposure to certain congeners with a wide range of potential non-carcinogenic adverse health effects, such as type 2 diabetes, respiratory, kidney, cardiovascular, neurological and degenerative diseases, osteoporosis, inflammation and

oxidative stress, increased rates of breast cancer, infertility, adverse pregnancy outcomes and other hormonal disorders, birth defects, neurocognitive and developmental disorders in childhood [20-29].

***Pesticides* -** Pesticides (from Latin “pestis” meaning plague, and “caedere” meaning to kill) are a wide range of chemicals used to manage and control pests (weeds, plant pathogens, insects, molluscs, nematodes, birds, fish, mammals and microbes) that compete with humans for food and can consequently destroy property and help carry or spread diseases or are seen as a bother. They are employed in numerous contexts, from agriculture (i.e., protecting crops from pests) to public health initiatives (i.e. control disease vectors such as mosquitoes). Pesticides can be divided according to their application (systemic or non-systemic, fumigants, repellent and, stomach poisoning), their function and what they are supposed to kill, and their chemical composition (OCPs, OPPs, carbamates, pyrethroid) [30].

Pesticides such as OCPs and OPPs, which are those most present in the considered two NPCSs, can cause acute and chronic health effects [30], including stinging eyes, rashes, and death, while chronic effects include cancers, such as Non-Hodgkin lymphoma and leukaemia [31-34], birth defects and reproductive harm [35,36], neurological effects as Parkinson’s and Alzheimer’s disease and cognitive impairment [30,37-39], respiratory problems such as asthma and chronic obstructive pulmonary diseases [40-44] and endocrine system disruption and cardiovascular diseases [45,46].

S3.2. Per- and PolyfluoroAlkyl Substances (PFAS)

PFAS are a group of over 4,700 widely used man-made chemicals that accumulate over time in living organisms and the environment, known as “permanent chemicals”. Due to their potential harm to the environment and health, they have become a focus of public health research [47]. Epidemiological studies have reported that human health effects due to environmental contamination by PFAS include infertility, steroid hormone disruption, thyroid, liver, and kidney disease, metabolic dysfunction, cardiovascular and reproductive system damage, and increased cancer risk [48-55].

S3.3. Polycyclic Aromatic Hydrocarbons (PAHs)

PAHs are hazardous chemicals composed exclusively of carbon and hydrogen, forming two or more aromatic rings linked in an angular, linear, or clustered arrangement. Although soluble in many organic solvents, they are typically insoluble in water, which contributes to their persistence in the environment, particularly in aquatic systems. Epidemiological studies on specific PAHs or their metabolites associate them with respiratory and cardiovascular diseases, osteoporosis, dyslipidemia, psychiatric disorders, inflammation and oxidative stress, metabolic syndrome, and susceptibility to cancer development [56-65].

S3.4. Volatile Organic Compounds (VOCs)

VOCs are organic compounds that evaporate from their liquid or solid form at room temperature, transforming into gases or vapors. Some VOCs are found in nature and are important for environmental interactions, while anthropogenic VOCs include a large group of chemicals derived from industrial processes, some of which are potentially hazardous to human health if inhaled at

certain concentrations. Numerous epidemiological studies have reported that the adverse health effects caused by exposure to these chemicals through ingestion, inhalation, or skin absorption are both short- and long-term: temporary irritation of the eyes or throat, nausea and headache, respiratory, cardiovascular, renal, liver, or central nervous system disorders, birth defects, leukemia, neurocognitive deficits, reproductive impairment, and cancer [66-79].

S3.5. Heavy metals

Heavy metals are natural components of the Earth's crust and persistent environmental pollutants, potentially hazardous to human health at low concentrations. They are produced both from natural sources, such as weathering of metalliferous rocks and volcanic eruptions, and from anthropogenic sources, such as mining and various industrial and agricultural activities, and dispersed into the environment. Heavy metals such as, Cd, As, Hg, Pb, Cr and aluminum are now recognized as systemic toxicants associated with various effects on human health, including chronic diseases such as cardiovascular disease, neurological, renal, pulmonary, and dermatological disorders, and various types of cancer [80-95].

S4. Biomarkers identified, treatment and analysis methodology

Table S1. Exposure biomarkers identified, treatment and analysis methodology for blood samples.

<table>
<tr><th colspan="4">Biological matrix – BLOOD</th></tr>
<tr><th colspan="2">Exposure biomarker selected</th><th>Sample Treatment</th><th>Biological Matrix Analysis Methodology</th></tr>
<tr><td>Non-dioxin-like polychlorinated biphenyls (NDL-PCBs)</td><td>PCB-28, PCB-52, PCB-101, PCB-138, PCB-153, PCB-180</td><td rowspan="3">• Collect a total of 15 mL of whole blood into specific tubes without anticoagulants
• Allow the blood to clot in the tube (approximately 4 hours) at room temperature
• Centrifuge for 15 minutes at approximately 3500 rpm at 4°C

Collect at least 7.5 mL of serum with a micropipette to avoid contamination and transferred to a glass tube

Freeze immediately after sampling at -20°C
Store the samples at the two collection centers.</td><td rowspan="3">Serum
High Resolution Gas Chromatography with tandem Mass Spectrometry (HRGC-MS/MS)</td></tr>
<tr><td>Organochlorine pesticides (OCPs)</td><td>HCH (alpha, beta, gamma - Isotopic Dilution), Hexachlorobenzene (Isotopic Dilution), Heptachlor (Isotopic Dilution), Aldrin (Isotopic Dilution), Dieldrin (Isotopic Dilution), cis- and trans-Chlordane (Isotopic Dilution), Endrin (Isotopic Dilution), DDT (p,p-DDE; p,p-DDD; p,p-DDT; o,p-DDT - Isotopic Dilution), Oxychlordane, Endosulfan alpha-, beta-, sulfate, Endrin, Heptachlor epoxide cis and trans, Quintozene, Tecnazene, Methoxychlor</td></tr>
<tr><td>Organophosphorus pesticides (OPPs)</td><td>Acefate, Azinfos Etile, Azinfos Metile, Bromofos, Bromophos-ethyl, Cadusafos, Carbofenothion, Chlorfenvinfos, Chlorpyrifos, Chlorpyrifos-methyl, Chlortiophos, Dialifos, Diazinone, Diclofenthion, Dimethoate, Disulfoton, Disulfoton Sulfossido, EPN, Ethion, Etopropos, Etrimfos, Fenamifos, Fenamifos Sulfone, Fenchlorfos, Fenchlorfos-oxon, Fenitrotion, Fenthion, Fenthoate, Forate, Formotion, Fosamidone, Fostiazate, Iodofenfos, Isocarbofos, Isofenofos, Isofenofos</td></tr>
</table>

	Metile, Malation, Metidation, Mevinfos, Parathion, Parathion-methyl, Phosalone, Pyrazofos, Pirimifos-ethyl, Pirimifos-methyl, Profenofos, Propetamfos, Protiofos, , Sulfotep, Terbufos, Tetrachlorvinphos, Thionazine, Triazofos, Vamidotion		
**Polychlorinated dibenzo-p-dioxins and dibenzofurans (PCDDs/PCDFs)**	2,3,7,8-TCDD, 1,2,3,7,8-PeCDD, 1,2,3,4,7,8-HxCDD, 1,2,3,6,7,8-HxCDD, 1,2,3,7,8,9-HxCDD, 1,2,3,4,6,7,8-HpCDD, OCDD, 2,3,7,8-TCDF, 1,2,3,7,8-PeCDF, 2,3,4,7,8-PeCDF, 1,2,3,4,7,8-HxCDF, 1,2,3,6,7,8-HxCDF, 1,2,3,7,8,9-HxCDF, 2,3,4,6,7,8-HxCDF, 1,2,3,4,6,7,8-HpCDF, 1,2,3,4,7,8,9-HpCDF, OCDF		Serum<br>High Resolution Gas Chromatography-High Resolution Mass Spectrometry (HRGC-HRMS) (Isotope Dilution)
**Dioxin-like polychlorinated biphenyls (DL-PCBs)**	PCB 77, PCB 81, PCB 126, PCB 169, PCB 105, PCB 114, PCB 118, PCB 123, PCB 156, PCB 157, PCB 167, PCB 189		
**Per- and polyfluoroalkyl substances (PFAS)**	Perfluorobutanoic acid, Perfluorobutanesulfonic acid, Perfluorododecanoic acid, Perfluoroheptanoic acid, Perfluorohexanoic acid, Perfluorohexanesulfonic acid, Perfluorononanoic acid, Perfluorooctanoic acid, Perfluorooctanesulfonic acid, Perfluoropentanoic acid, Perfluoroundecanoic acid	• Collect a total of 5 mL of whole blood into designated tubes without anticoagulants<br>• Place the tube in a special rack in a vertical position (avoiding any stress on the contents as much as possible) and leave it at room temperature until the clot has completely formed (approximately 30 minutes)<br>• Centrifuge the blood sample within 2 hours of collection at 3500 rpm for 15 minutes at 20°C<br>• Transfer the serum obtained using a glass pipette into a polypropylene tube with a screw cap (13 x 100 mm, 15 mL tubes)<br>Freeze the serum tubes at -20°C and keep the tube upright.<br>Store the samples at the two collection centers.	Serum<br>High Performance Liquid Chromatography with tandem Mass Spectrometry (HPLC MS/MS)
**Heavy metals**	Chromium (Cr), Lead (Pb) and Copper (Cu)	For whole blood samples (for the analysis of Cr and Pb)<br>• Collect 3 mL of whole blood in tubes with purple EDTA caps, with anticoagulants<br>• Do not centrifuge or open<br>• Freeze at -20°C<br>For serum samples (for the analysis of Cu)<br>• Collect 3.5 mL of whole blood in tubes without anticoagulants, with yellow caps and gel separator<br>• Centrifuge for 15 minutes at 3500 rpm at 4°C after the clot has retracted<br>• Collect at least 1 mL of serum and transfer it to a cryogenic tube.<br><br>Freeze the product at -20°C.<br>Store the samples at the two collection centers.<br>If samples are transferred to the laboratory weekly, they can be stored in the refrigerator.	Whole blood and serum<br>Inductively Coupled Plasma Mass Spectrometry (ICPMS)

Table S2. Exposure biomarkers identified, treatment and analysis methodology for urine samples.

Biological matrix – BLOOD			
**Exposure biomarker selected**		**Sample Treatment**	**Biological Matrix Analysis Methodology**
**Non-dioxin-like polychlorinated biphenyls (NDL-PCBs)**	PCB-28, PCB-52, PCB-101, PCB-138, PCB-153, PCB-180	• Collect a total of 15 mL of whole blood into specific tubes without anticoagulants<br>• Allow the blood to clot in the tube (approximately 4 hours) at room temperature<br>• Centrifuge for 15 minutes at approximately 3500 rpm at 4°C<br><br>Collect at least 7.5 mL of serum with a micropipette to avoid contamination and transferred to a glass tube<br><br>Freeze immediately after sampling at -20°C<br>Store the samples at the two collection centers.	Serum<br>High Resolution Gas Chromatography with tandem Mass Spectrometry (HRGC-MS/MS)
**Organochlorine pesticides (OCPs)**	HCH (alpha, beta, gamma - Isotopic Dilution), Hexachlorobenzene (Isotopic Dilution), Heptachlor (Isotopic Dilution), Aldrin (Isotopic Dilution), Dieldrin (Isotopic Dilution), cis- and trans-Chlordane (Isotopic Dilution), Endrin (Isotopic Dilution), DDT (p,p-DDE; p,p-DDD; p,p-DDT; o,p-DDT - Isotopic Dilution), Oxychlordane, Endosulfan alpha-, beta-, sulfate, Endrin, Heptachlor epoxide cis and trans, Quintozene, Tecnazene, Methoxychlor		
**Organophosphorus pesticides (OPPs)**	Acefate, Azinfos Etile, Azinfos Metile, Bromofos, Bromophos-ethyl, Cadusafos, Carbofenothion, Chlorfenvinfos, Chlorpyrifos, Chlorpyrifos-methyl, Chlortiophos, Dialifos, Diazinone, Diclofenthion, Dimethoate, Disulfoton, Disulfoton Sulfossido, EPN, Ethion, Etopropos, Etrimfos, Fenamifos, Fenamifos Sulfone, Fenchlorfos, Fenchlorfos-oxon, Fenitrotion, Fenthion, Fenthoate, Forate, Formotion, Fosamidone, Fostiazate, Iodofenfos, Isocarbofos, Isofenofos, Isofenofos Metile, Malation, Metidation, Mevinfos, Parathion, Parathion-methyl, Phosalone, Pyrazofos, Pirimifos-ethyl, Pirimifos-methyl, Profenofos, Propetamfos, Protiofos, , Sulfotep, Terbufos, Tetrachlorvinphos, Thionazine, Triazofos, Vamidotion		
**Polychlorinated dibenzo-p-dioxins and dibenzofurans (PCDDs/PCDFs)**	2,3,7,8-TCDD, 1,2,3,7,8-PeCDD, 1,2,3,4,7,8-HxCDD, 1,2,3,6,7,8-HxCDD, 1,2,3,7,8,9-HxCDD, 1,2,3,4,6,7,8-HpCDD, OCDD, 2,3,7,8-TCDF, 1,2,3,7,8-PeCDF, 2,3,4,7,8-PeCDF, 1,2,3,4,7,8-HxCDF, 1,2,3,6,7,8-HxCDF, 1,2,3,7,8,9-HxCDF, 2,3,4,6,7,8-HxCDF, 1,2,3,4,6,7,8-HpCDF, 1,2,3,4,7,8,9-HpCDF, OCDF		Serum<br>High Resolution Gas Chromatography-High Resolution Mass Spectrometry (HRGC-HRMS) (Isotope Dilution)
**Dioxin-like polychlorinated biphenyls (DL-PCBs)**	PCB 77, PCB 81, PCB 126, PCB 169, PCB 105, PCB 114, PCB 118, PCB 123, PCB 156, PCB 157, PCB 167, PCB 189		
**Per- and polyfluoroalkyl substances (PFAS)**	Perfluorobutanoic acid, Perfluorobutanesulfonic acid, Perfluorododecanoic acid, Perfluoroheptanoic acid, Perfluorohexanoic acid, Perfluorohexanesulfonic acid, Perfluorononanoic acid, Perfluorooctanoic acid, Perfluorooctanesulfonic acid,	• Collect a total of 5 mL of whole blood into designated tubes without anticoagulants<br>• Place the tube in a special rack in a vertical position (avoiding any stress on the contents as much as possible) and leave it at room temperature until the clot has completely formed (approximately 30 minutes)	Serum<br>High Performance Liquid Chromatography with tandem Mass Spectrometry (HPLC MS/MS)

	Perfluoropentanoic acid, Perfluoroundecanoic acid	• Centrifuge the blood sample within 2 hours of collection at 3500 rpm for 15 minutes at 20°C<br>• Transfer the serum obtained using a glass pipette into a polypropylene tube with a screw cap (13 x 100 mm, 15 mL tubes)<br>Freeze the serum tubes at -20°C and keep the tube upright.<br>Store the samples at the two collection centers.	
**Heavy metals**	Chromium (Cr), Lead (Pb) and Copper (Cu)	For whole blood samples (for the analysis of Cr and Pb)<br>• Collect 3 mL of whole blood in tubes with purple EDTA caps, with anticoagulants<br>• Do not centrifuge or open<br>• Freeze at -20°C<br>For serum samples (for the analysis of Cu)<br>• Collect 3.5 mL of whole blood in tubes without anticoagulants, with yellow caps and gel separator<br>• Centrifuge for 15 minutes at 3500 rpm at 4°C after the clot has retracted<br>• Collect at least 1 mL of serum and transfer it to a cryogenic tube.<br><br>Freeze the product at -20°C.<br>Store the samples at the two collection centers.<br>If samples are transferred to the laboratory weekly, they can be stored in the refrigerator.	Whole blood and serum Inductively Coupled Plasma Mass Spectrometry (ICPMS)
Creatinine will also be determined in the urine sample, both to express the results of biomarkers eliminated by glomerular filtration (1-hydroxypyrene, mercury, arsenic and, metals) in μg/g creatinine and to account for sample dilution. For each biomarker, the quantitative assay will be performed against a calibration curve constructed in the concentration			

Table S3. Selected analytes and respective methodologies for clinical biochemistry analysis of blood samples, the description, the tube type, and the storage method are also provided.

**Biological matrix – BLOOD**		
**Description of the analytes selected**	**Test Tube Type/ Sample Treatment**	**Analysis methodology**
Creatinine [Serum/Plasma]	Collect a total of 20.7 mL of whole blood divided in:<br>2 x PET microlyzed silica acrylic gel tube, 13x100, 5.0 mL aspirating volume; yellow cap. Ref. 367955 (total volume 10 mL)<br>1 x PET EDTA K2 5.4 mg coating tube, 13x75, 3.0 mL aspirating volume; lilac cap. Ref. 364664<br>1 x PET EDTA K2 tube, 5.4 mg, Coating 13x75, aspiration volume 3.0 mL transparent lilac cap<br>Ref. 368856<br>1x PET/PP tube Sodium Citrate (0.109M, 3.2%) with blue cap; 13x75 aspiration volume 2.7 mL<br>Ref. 363048	Flow cytometry, enzymatic/colorimetric assay, capillary electrophoresis, electrochemiluminescence assay (ECLIA)
Sodium [Serum/Plasma]		
Potassium [Serum/Plasma]		
Chlorine [Serum/Plasma]		
Inorganic phosphate		
Total Calcium [Serum/Plasma]		
Creatine Phosphokinase (CPK or CK) [Serum/Plasma]		
Lactate DeHydrogenase [LDH] [Serum/Plasma]		
Red Blood Cell Distribution Width (RDW)		
Mean Corpuscular Haemoglobin Concentration (MCHC)		
Mean Haemoglobin Content (MCH)		
Mean Corpuscular Volume (MCV)		
Haematocrite (HCT)		
Haemoglobin (HGB)		

Erythrocytes (RBC)	1x PET tube Sodium Fluoride (2.5 mg/mL) / Potassium Oxalate (2.0 mg/mL) 13X75 aspiration volume 2.0 mL gray cap<br>Ref. 368920<br><br>First collect samples in a citrate tube (blue cap), then in a serum tube (yellow cap) with a separator gel, then in an EDTA tube, and finally in a grey-cap tube.<br>• Mix each tube by inverting (4-8 inversions per tube; do not shake).<br>• Do not centrifuge or pre-treat the samples.<br>• Store at room temperature.<br><br>Send immediately to the reference laboratory.	
Leukocytes (WBC)		
Neutrophils		
Lymphocytes		
Monocytes		
Eosinophils		
Basophils		
Platelets		
Glucose [serum/plasma]		
Total protein		
Iron [Serum/Plasma]		
Ferritin [Serum/Plasma]		
Transferrin [Serum/Plasma] S		
Cholinesterase (Pseudo-Che) [Serum/Plasma]		
Albumin [Serum/Plasma]		
Alpha-1 globulin		
Alpha-2 globulin		
Beta-1 globulin		
Beta-2 globulin		
Direct Bilirubin		
Total bilirubin		
Alkaline Phosphatase [Serum/Plasma]		
Estradiol (E2) [Serum/Plasma]		
Follitropin or Follicle-Stimulating Hormone (FSH) [Serum/Plasma]		
Luteotropin or Luteinizing hormone [LH] [Serum/Plasma]		
Prolactin [Serum/Plasma]		
Total Testosterone [Serum/Plasma]		
Free Testosterone [Serum/Plasma]		
Cortisol [Serum/Plasma]		
Thyroglobulin Antibodies (anti-Tg) [Serum/Plasma]		
Thyroid Peroxidase Antibodies (anti-TPO) [Serum/Plasma]		
Free Triiodothyronine (FT3) [Serum/Plasma]		
Free Thyroxine (FT4) [Serum/Plasma]		
Thyrotropin or Thyroid-Stimulating Hormone (TSH) [Serum/Plasma]		
Homocysteine [serum/plasma]		
Insulin [Serum/Plasma]		
25-OH Vitamin D [Serum/Plasma]		
Alpha-1 Fetoprotein (AFP)		
Aspartate Aminotransferase (AST)		
Alanine Aminotransferase (ALT)		
Urea [Serum/Plasma]		

Gamma-glutamyl transpeptidase (γ-GT) [Serum/Plasma]		
Uric Acid [Serum/Plasma]		
Estimated Glomerular Filtration Rate (EGFR)		
Glycated haemoglobin (HbA1c)		
Triglycerides [Serum/Plasma]		
Total Cholesterol [Serum/Plasma]		
High Density Lipoprotein Cholesterol (HDL) [Serum/Plasma]		

Table S4. Cardiovascular and renal risk biomarkers identified, treatment and analysis methodology for blood and urine samples. Characteristics of instrumental measurements are also reported.

Biological matrix - BLOOD			
**Cardiovascular and renal risk biomarkers selected**		**Sample Treatment**	**Analysis methodology**
**Inflammatory status**	*High sensitivity CRP, interleukin-6, interleukin-18, interleukin-1β, Tumor Necrosis Factor, Monocyte Chemoattractant Protein 1*	For <u>plasma samples</u>:<br>• Collect 10 mL of whole blood in an EDTA tube with lilac cap<br>• Centrifuge the tubes for 15 minutes, 3500 rpm, at 4°C<br>• Transfer the plasma into 2 mL Eppendorf tubes in 500 μL aliquots<br><br>For <u>serum samples</u>:<br>• Collect 5 mL of whole blood in serum tubes with yellow caps (with clotting activator)<br>• Store upright at room temperature and separate within 1 hour.<br>• Centrifuge the tubes for 15 minutes, 3500 rpm, at 4°C<br>• Transfer the serum into 2 mL Eppendorf tubes in 500 μL aliquots<br><br>Freeze the plasma and serum aliquots at -20°C.<br>Store the samples at the two collection centers.	Milliplex analysis
**Oxidative stress**	*F2 isoprostanes, 3-nitrotyrosine, thiobarbituric acid reactive substances (TBARS), 4-hydroxynonenal*		ELISA assay and spectrophotometric methods
**Renal impairment**	*cystatin C, beta-2 microglobulin*		
**Biological matrix - URINE**			
**Renal impairment**	*Albuminuria, creatininuria and their ratio, KIM-1, NGAL, beta-2 microglobulin*	• Collect 120 mL of the first urine of the morning using a sterile collection tube<br>• Midstream collection: collect the middle of the urinary stream, discarding the first 20 mL and collecting the next 30 mL<br>• Centrifuge the urine at 2000-3000 rpm for 10 minutes and collect the supernatant (the liquid portion)<br><br>If the sample must be stored for less than a month before being sent to the laboratory, aliquot into six 500 μL tubes and store at -20°C<br>If long-term storage and preservatives are required, aliquot into six 500 μL tubes and store at -80°C.<br>Store the samples at the two collection centers.	ELISA assay and spectrophotometric methods
**INSTRUMENTAL MEASUREMENTS**			
**Early vascular remodeling and damage**	*Cardiac age, Pulse Wave Analysis (PWA), central Blood Pressure (cBP), Augmentation index (Aix), Cardiac Output (CO), Total Peripheral vascular Resistance (TPR)*	<u>*1. Acquisition of the 12-lead ECG signal using the Cardioline TouchECG HD12 device*</u><br>• Place the subject in a supine position and wait at least 3 minutes of rest<br>• Attach the electrodes to the subject's chest, following the manufacturer's instructions<br>• If the subject has hairy chest, remove any hair with a razor<br>• Enter the subject's data into the app (only the study ID and not any sensitive information)<br>• Wait until the ECG signal is stable<br>• Ask the subject not to move during the recording	*12-lead ECG;*<br>*BP+ system*

		• Start recording the waveform and save at least 2 minutes of stable waveforms in the dedicated app.<br>• acquire and save the ECG signal using the dedicated app.<br>*2. Acquisition of the central and brachial blood pressure waveform using the BP+ system*<br>• Place the blood pressure cuff on the subject's forearm<br>• Measure blood pressure by pressing the start button on the portable device connected<br>to the blood pressure cuff<br>• Compile a study report relating to the blood pressure readings	

Table S5. Respiratory disease risk biomarkers identified, treatment and methodology for saliva samples.

Biological matrix - SALIVA		
**Biomarkers of respiratory diseases risk**	**Sample Treatment**	**Analysis methodology**
**Reactive Oxygen Species (ROS)**	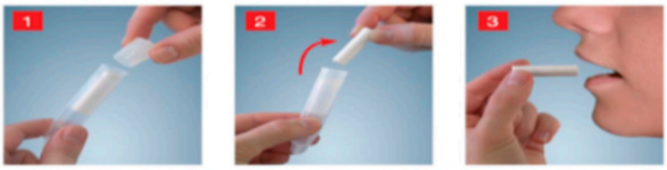	Electron Paramagnetic Resonance (EPR) spectroscopy,
**Oxidative DNA damage**<br>*8-hydroxydeoxyguanosine*		ELISA kit
**Cortisol concentration**	The subjects remove the swab from the Salivette® devices (CE-marked medical devices manufactured by Sarstedt, Nümbrecht, Germany) (Figure 1 and 2) and places the swab into the mouth and chews it for about 90 seconds to stimulate salivation (Figure 3)<br>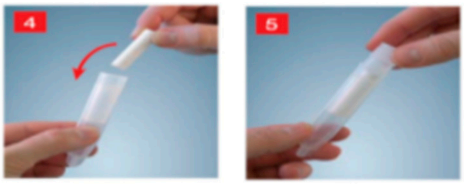	
**Carbonic anhydrase 6 (CA6)**	Now the subjects return the swab with the absorbed saliva to the Salivette (Figure 4), and replace the stopper (Figure 5)<br><br>Centrifugate at 1500 g for 2-5 minutes at 4°C<br>Transfer the recovered saliva (approximately 1 mL) into aliquots<br>Freeze samples at -80°C in the two study centers	

Table S6. Disease risk biomarkers and biological aging indicators identified, treatment and analysis methodology for blood samples.

Biological matrix - BLOOD		
**Diseases risk biomarker and biological aging indicators**	**Treatment**	**Analysis methodology**
Telomere length	• Collect 3 mL of whole blood in an EDTA tube with lilac cap<br>• divide the whole blood into two cryogenic tubes with aliquots of 500 µL<br><br>Freeze immediately after sampling at -20°C or -80°C	• DNA will be extracted from whole blood aliquoted in cryogenic tubes using the QIAamp® DNA Blood Mini Kit (QIAGEN) (Qiagen)<br>• DNA concentration and quality will be assessed using a NanoDrop Lite spectrophotometer (Thermo Scientific)<br>• TL will be analyzed in triplicate using real-time qRT-PCR on a Bio-Rad CFX384 system, with a negative control included in each plate.

Global DNA methylation	Store the samples at the two collection centers.	• DNA will be extracted from whole blood aliquoted in cryogenic tubes using the QIAamp® DNA Blood Mini Kit (QIAGEN) (Qiagen)<br>• DNA samples will be quantified using the Qubit 4.0 fluorimeter (Invitrogen) with the dsDNA HS assay kit (Invitrogen).<br>• The identification of differentially methylated CpG sites between sample groups will be performed using the Illumina Infinium Methylation Screening Array (MSA) platform.

Table S7. Treatment and analysis methodology for blood samples for metabolomic profiling.

Biological matrix - BLOOD		
**Metabolomic profile descriptors**	**Sample Treatment**	**Analysis methodology**
Fatty acids, amino acids, Krebs cycle intermediates, organic acids and ketoacids, alpha- and beta-hydroxybutyrate, glycerate, and uric acid etc.	• Collect 3 mL of whole blood in an EDTA tube with lilac cap<br>• Keep samples at a low temperature (for example, in a thermal container with refrigerated packs)<br>• Centrifuge samples within one hour of collection for 15 minutes, 3500 rpm, at 4°C<br>• Collect the plasma (supernatant) and collect a 500 µL aliquot in tubes suitable for cryopreservation.<br><br>Freeze the sample at -20°C or -80°C.<br>Store the samples at the two collection centers.	• Collect approximately 50-60 µL of plasma and add 20 µL of internal standards mix labelled with stable isotopes for targeted quantification (MSK-A2-S Metabolomics Amino Acid Standard Mix, CIL Cambridge, MA, USA and MSK-OA-1 Labeled Organic Acid Mix, CIL Cambridge, MA, USA)<br>• Before analysis, precipitate the proteins by centrifugation after adding 200 µL of cold methanol<br>• Dry the supernatant under nitrogen and methoxylate it with 10 µL of a 20 mg/ mL methoxylamine solution for 30 minutes at 60°C<br>• After evaporation to dryness under nitrogen, derivatize the sample using 30 µL of N-tert-butyldimethylsilyl-N-methyltrifluoroacetamide (TBDMS, Merck, Germany) or N,O-Bis(trimethylsilyl)trifluoroacetamide with Trimethylchlorosilane (BSTFA-TMCS 99:1, Merck, Germany) with the addition of 70 µL of acetonitrile (1 hour at 60°C)<br>• Transfer the volume to vials for analysis by gas chromatography-mass spectrometry (GCMS 5975 and GC-QQQ 8890/7000, Agilent Technologies, Santa Clara, CA, USA) equipped with a DB5-MS column (30 m × 0.25 mm i.d., 0.25 µm) (Agilent Technologies, Santa Clara, CA, USA)<br><br>Agilent software will be used for peak identification and metabolite measurement. NIST and Fiehn libraries will be used for compound identification in untargeted analyses, while the concentration of targeted metabolites will be quantified using labeled internal standards (see above).<br><br>Total free fatty acid concentration will be determined spectrophotometrically on an EDTA plasma sample (25 µL) using a validated kit (Non-Esterified Fatty Acid (NEFA) Assay Kit, FujiFilm Wako, USA)

Table S8. Procedure for collecting blood samples to be stored in the biobank.

**Biological matrix - BLOOD**
Procedure:<br>• Collect 6 mL of whole blood in EDTA tubes (lilac caps)<br>• Transfer 1 mL of whole blood to cryogenic tubes<br>• Centrifuge the remaining 5 mL within one hour of collection for 15 minutes, 3500 rpm, at 4°C to obtain approximately 1 mL of plasma (supernatant)<br>• Transfer the supernatant to 2 mL cryogenic tubes<br><br>• Collect 3.5 mL of whole blood in tubes without anticoagulant<br>• Keep the samples at room temperature for 30 to 45 minutes to promote clot formation<br>• Centrifuge at room temperature 15 minutes, at 3500 rpm, at 4°C<br>• Transfer the supernatant (serum) obtained to cryogenic tubes<br><br>Freeze the sample at -20°C or -80°C.<br>Store the samples at the two collection centers.

Table S9. Distribution by municipality, sex, age group and exposure of the subjects to be recruited and the subjects extracted from the Registry of Assisted Persons.

			**Livorno/Collesalvetti**		**Piombino**	
**Sex**	**Age class**	**Exposure class**	**Number of extracted subjects**	**Number of subjects to obtain**	**Number of extracted subjects**	**Number of subjects to obtain**
M	20-34	1	208	35	119	19
		2	170	28	22	4
		3	110	18	65	11
		4	38	6	36	6
F	20-34	1	185	31	111	18
		2	167	28	22	4
		3	98	16	57	9
		4	36	6	9	6
M	35-49	1	284	47	172	27
		2	232	39	28	5
		3	137	23	77	13
		4	54	9	10	2
F	35-49	1	276	46	165	27
		2	250	42	32	5
		3	146	24	77	13
		4	52	9	2	2
M	50-64	1	311	52	216	34
		2	282	47	43	7
		3	185	31	131	22
		4	68	11	5	1
F	50-64	1	325	54	223	35
		2	309	52	46	8
		3	205	34	130	21
		4	71	12	2	1
		**TOTAL**	**4200**	**700**	**1800**	**300**

1.
